# Contribution of the Microbiome, Environment, and Genetics to Mucosal Type 2 Immunity and Anaphylaxis in a Murine Food Allergy Model

**DOI:** 10.3389/falgy.2022.851993

**Published:** 2022-03-31

**Authors:** Kelsey G. Stark, Nicole R. Falkowski, Christopher A. Brown, Roderick A. McDonald, Gary B. Huffnagle

**Affiliations:** ^1^Mary H. Weiser Food Allergy Center, University of Michigan, Ann Arbor, MI, United States; ^2^Department of Internal Medicine, Division of Pulmonary and Critical Care Medicine, University of Michigan, Ann Arbor, MI, United States; ^3^Department of Molecular, Cellular, and Developmental Biology, University of Michigan, Ann Arbor, MI, United States; ^4^Institute for Research on Innovation and Science (IRIS), Institute for Social Research (ISR), University of Michigan, Ann Arbor, MI, United States

**Keywords:** *Candida albicans*, Balb/c, mast cell, IL-6, *Lachnospiraceae*, food allergy, microbiome, Th2

## Abstract

There is heterogeneity inherent in the immune responses of individual mice in murine models of food allergy, including anaphylaxis, similar to the clinical heterogeneity observed in humans with food allergies to a defined food. One major driver of this heterogeneity may be differences in the microbiome between sensitized individuals. Our laboratory and others have reported that disruption of the microbiome (dysbiosis) by broad spectrum antibiotics and/or yeast colonization can alter systemic immunity and favor the development of mucosal Type 2 immunity to aeroallergens. Our objective was to use a well-characterized murine model (Balb/c mice) of food allergies (chicken egg ovalbumin, OVA) and determine if antibiotic-mediated dysbiosis (including *C. albicans* colonization) could enhance the manifestation of food allergies. Furthermore, we sought to identify elements of the microbiome and host response that were associated with this heterogeneity in the anaphylactic reaction between individual food allergen-sensitized mice. In our dataset, the intensity of the anaphylactic reactions was most strongly associated with a disrupted microbiome that included colonization by *C. albicans*, loss of a specific *Lachnoclostridium* species (tentatively, *Lachnoclostridium* YL32), development of a highly polarized Type 2 response in the intestinal mucosa and underlying tissue, and activation of mucosal mast cells. Serum levels of allergen-specific IgE were not predictive of the response and a complete absence of a microbiome did not fully recapitulate the response. Conventionalization of germ-free mice resulted in *Akkermansia muciniphila* outgrowth and a higher degree of heterogeneity in the allergic response. C57BL/6 mice remained resistant even under the same dysbiosis-inducing antibiotic regimens, while changes in the microbiome markedly altered the reactivity of Balb/c mice to OVA, as noted above. Strikingly, we also observed that genetically identical mice from different rooms in our vivarium develop different levels of a Type 2 response, as well as anaphylactic reactions. The intestinal microbiome in these mice also differed between rooms. Thus, our data recapitulate the heterogeneity in anaphylactic reactions, ranging from severe to none, seen in patients that have circulating levels of food allergen-reactive IgE and support the concept that alterations in the microbiome can be one factor underlying this heterogeneity.

## Introduction

Food allergy prevalence has increased dramatically over the past few decades and affects ~6.5% of children under the age of 18 (4.8 million) (1–3). Food allergy is defined as “an adverse health effect arising from a specific immune response that occurs reproducibly on exposure to a given food” according to the NIAID (4, 5). The most common food allergens are peanut, tree nuts, chicken egg, cow's milk, fish, shellfish, soybean, wheat, and sesame (6, 7). Following ingestion of a food allergen, there is a spectrum of possible reactions that can affect the gastrointestinal tract, respiratory tract, skin and circulatory system, which can range from mild to life-threatening (5, 6, 8–10). Currently, there are no serologic or immunologic prognostic criteria for identifying individuals who will develop food allergen-induced anaphylaxis, and controlled food challenge tests are the only option available for a definitive diagnosis of a severe food allergy.

Individuals with food allergies typically develop Type 2 responses (11) to the allergen, including the production of a number of pro-inflammatory cytokines that drive allergic responses, such as interleukin (IL)-4, IL-5, IL-9, and IL-13 from Th2 and ILC2 cells. In IgE-mediated food allergies, IL-4 and IL-13 can promote allergen-specific immunoglobulin E (IgE) antibody production and intestinal allergic inflammation (12–16). Both IL-4 and IL-13 also influence the recruitment and activation of various innate immune cells such as intestinal mast cells, eosinophils, and alternatively activated (M2a) macrophages (8, 13, 17–20). M2a macrophages are activated by these cytokines through the shared common subunit receptor, IL-4Rα (18–20). Upon alternative activation, Ym1 (chitinase-like protein 3), Arginase-1, CCL11 and CCL24 are expressed (18–22). IL-5 and GM-CSF are produced by a number of cell types involved in allergic disease such as mast cells, activated Th2 cells and ILC2, and these cytokines stimulate eosinophil proliferation and continued survival (23–25).

Mast cells are one of the major effector cells of allergic disease; they are found throughout the body at baseline and are increased during allergic responses (26, 27). IL-9-producing mucosal mast cell precursors have been identified, and increased numbers of these cells are drivers of IgE-mediated experimental food allergy (28). Resident mucosal mast cells (MMCs) are also activated by IL-9 which can promote intestinal mastocytosis and food allergy through immunoglobulin ε receptor (FcεR1)-bound IgE in a mouse model (8, 14, 26–30). Mice that transgenically over-express IL-9 *via* an intestine-specific promoter develop robust food allergic responses in an animal model (14, 29, 30). Conversely, in the absence of IL-9 or IL-9R, repeated oral challenge with a food allergen (ovalbumin, OVA) makes mice produce OVA-specific IgE and IgG, but lack other parameters associated with a food allergic response such as hypothermia, intestinal mastocytosis, and increased serum levels of mast cell protease-1 (MCPT-1) (30). MCPT-1, MCPT-4 and MCPT-6 are produced by different types of mast cells, with MMCs expressing MCPT-1 and connective tissue mast cells (CTMCs) expressing MCPT-4 and −6 (28, 31, 32). Despite the extensive work that has been done on mucosal allergic responses to food allergens, there remains a significant heterogeneity in the anaphylactic responses amongst food allergen-sensitized individuals that has yet to be explained.

In our current study, one of our objectives was to address potential variables that contribute to the heterogeneity of the anaphylactic response using a previously published model of food allergen-induced anaphylaxis in Balb/c mice (28–30). In this model, mice are primed by intraperitoneal injection of chicken egg protein, ovalbumin (OVA), mixed with alum and then subsequently challenged by repeated oral gavage with the allergen. Investigators have previously demonstrated that the number of IL-9 producing MMCs increase in the jejunum, and the development of IL-13^+^IFNγ^−^CD4+ T cells is positively correlated with the severity of anaphylaxis following oral antigen challenge (28). The anaphylactic response requires OVA-specific IgE activation of MMCs through FcεR1, with no demonstrable role for OVA-specific IgG1 in the intestinal response (28, 30). In sharp contrast, C57BL/6 mice, which are more commonly used for the study of mucosal immunity, do not develop systemic anaphylaxis in response to sensitization/challenge with OVA. This correlates strongly with fewer IL-9 producing MMCs in the jejunum, fewer IL-13^+^IFNγ^−^CD4+ T cells, lower serum MCPT-1 levels after challenge, and lower serum levels of OVA-specific IgE and IgG1 in C57BL/6 mice (28).

Our second objective was to determine if induction of intestinal dysbiosis could augment the food allergy response in this murine model. Previously published studies from our lab and others have demonstrated that transient disruption of the bacterial microbiome by broad-spectrum antibiotics and subsequent low level long-term intestinal colonization of mice by the yeast *Candida albicans* can result in enhanced susceptibility to the induction of airway allergies in these mice (33–35). In addition, *C. albicans* colonization can alter the re-colonization kinetics of the bacterial microbiome in the gastrointestinal tract after broad spectrum antibiotic treatment (36–38). A previous report has suggested that gastrointestinal colonization by *C. albicans* can affect food allergy sensitization in mice (39) and alter mast cell numbers in the mucosa (40). Thus, our objective was to use the previously described murine model of food allergy and determine if a similar induction of dysbiosis (including *C. albicans* colonization) also enhances the development or manifestation of a food allergy and anaphylaxis.

## Materials and Methods

### Murine Model of Food Allergy

**Figure 1:**
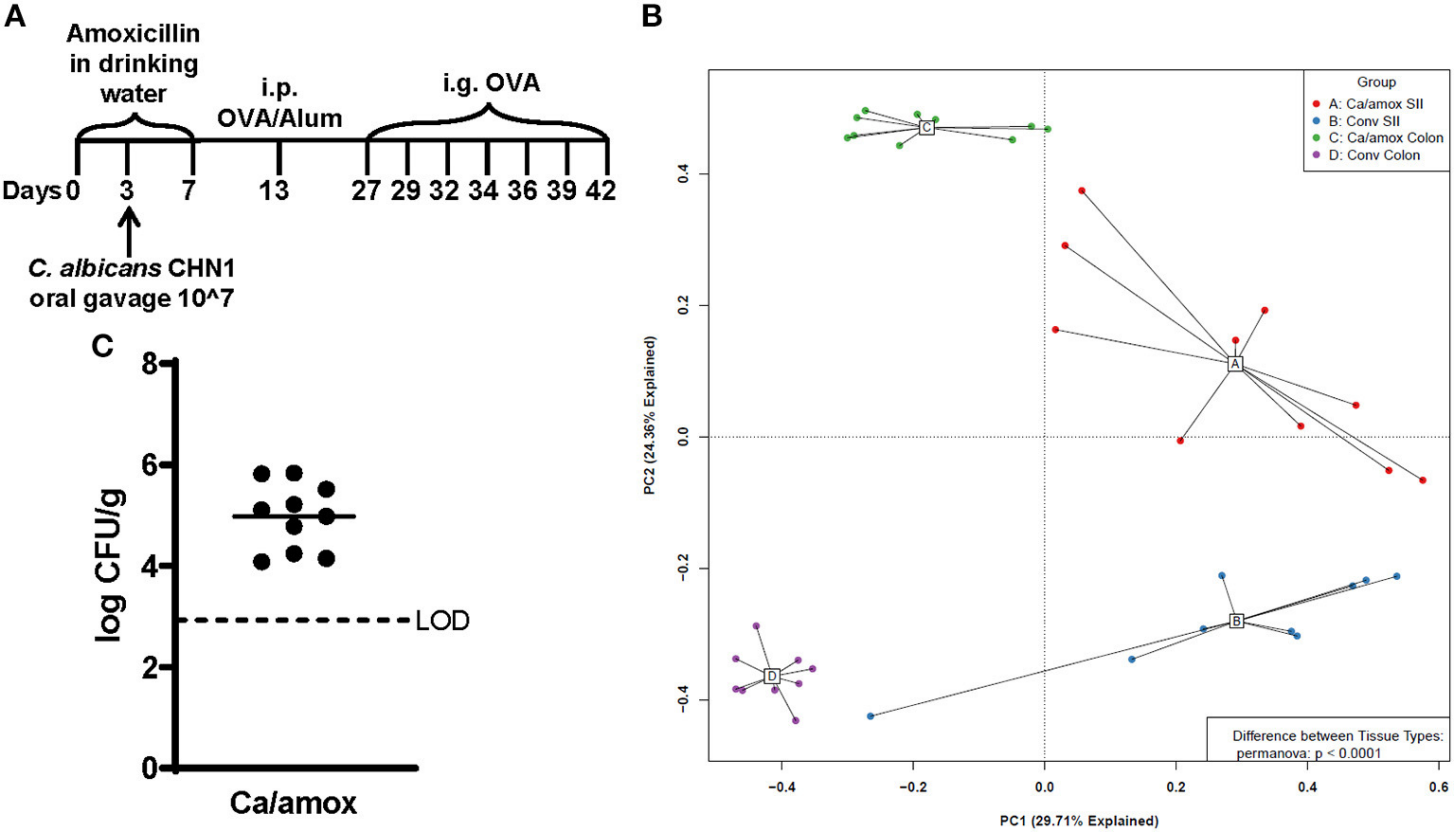
Experimental model and initial composition of microbiome and *Candida albicans* CHN1 in conventional (Conv) and microbiota disrupted (Ca/amox) Balb/cJ mice. **(A)** Mice in the Ca/amox group were given amoxicillin in their drinking water for 7 days prior to the initial allergen sensitization. On day 3 of antibiotic administration, *C. albicans* strain CHN1 was orally given *via* intragastric gavage (i.g.) at a concentration of 10^7^. On day 13, Conv and Ca/amox mice were intraperitoneally (i.p.) sensitized with 50 μg endotoxin free ovalbumin and subsequently challenged *via* i.g. administration with ovalbumin (OVA) dissolved in sterile saline. Each oral challenge occurred after a 3-4 h fast every 2 or 3 days for a total of seven complete challenges. Samples were collected for the groups of mice, untreated (Naive) and treated groups (Conv and Ca/amox) after challenge seven on day 42. The measurements included body temperature (0-2 h), serology, gastrointestinal snips for RT-qPCR and **(B)** microbiome principal component analysis, and **(C)** CFU of *C. albicans* from the cecal contents. A single dot represents one mouse; *n* = 9 to 10 mice. LOD is the limit of detection. Statistical significance was determined using PERMANOVA test. Statistical significance is *P* < 0.05. Small intestine ileum (SII).

All mice used in these experiments were 8-10 week old female Balb/cJ, C57BL/6J or germ-free Balb/c mice (GF). Balb/cJ and C57BL/6J mice were acquired from Jackson Laboratory (Bar Harbor, ME). They were cared for under specific-pathogen-free conditions at the University of Michigan (Ann Arbor, MI) following the guidelines outlined by the Institutional Animal Care and Use Committee (IACUC). GF mice were bred and cared for in the Germ-free Facilities at the University of Michigan (Ann Arbor, MI) following strict guidelines for maintaining a germ-free status as outlined by IACUC. Figures 1A, **4A** depict the timeline for sensitization and elicitation of food allergy in this mouse model. Day 0 sensitization of the mice consisted of an intraperitoneal (i.p.) injection with 50 ug endotoxin free (InvivoGen, San Diego, CA) ovalbumin (OVA) with 1 mg aluminum (alum) adjuvant. At day 14 post-sensitization, the mice were first fasted for 3-4 h prior to administration of an intragastric (i.g.) infusion of 50 mg of OVA (Sigma-Aldrich, Darmstadt, Germany) dissolved in sterile saline. The i.g. challenge was performed with a feeding needle and repeated every 2-3 days for a total of seven oral challenges. Baseline rectal temperatures were taken prior to the seventh OVA challenge and post-challenge; body temperatures were recorded every 15 min with a rectal probe (Physitemp Instruments, Clifton, New Jersey). The mice were subsequently euthanized, and blood was collected as well as tissue samples from the stomach, small intestine, cecum, and colon. Each tissue section was placed in RNAlater (ThermoFisher Scientific, Waltham, MA) and stored at −20°C for 24 h and then transferred to −80°C until processed (29).

### ELISA Measurements

Serum levels of mouse MCPT-1 were evaluated by ELISA (ThermoFisher Scientific, Vienna, Austria and BD Biosciences, San Diego, CA, respectively). OVA-specific IgG1, IgG2a, and IgE in the serum were measured by ELISA, according to the following protocol. Plates were coated with OVA in BupH™ Carbonate-Bicarbonate buffer (ThermoFisher Scientific, Vienna, Austria), pH 9.4 and incubated at 4°C overnight. The plates were washed with 1x TBS 0.05% Tween-20 buffer and blocked with “Blocker Casein” in TBS (ThermoFisher Scientific, Vienna, Austria) at room temperature for 2 h. Serial dilutions of the mouse anti-ova IgG1, anti-ova IgG2a or anti-ova IgE standards (Chondrex, Woodinville, WA) were prepared. After dilution of experimental serum samples, the diluted standards and samples were added to the plate in duplicate and incubated for 1 h. Subsequently, the plates were washed and goat anti-mouse IgG1-horseradish peroxidase (HRP) (Abcam, Cambridge, UK) or goat anti-mouse IgG2a-HRP (Abcam, Cambridge, UK) or goat anti-mouse IgE-HRP (Bio-Rad, Hercules, CA) were added to appropriate wells. After incubation, TMB substrate was added to each well and incubated covered with foil until developed. Reactions were stopped with H_2_SO_4_, and the optical density of the reactions was measured on an ELISA plate reader at 450 nm.

### RNA Isolation and Expression Analysis

RNA was isolated and purified from the appropriate tissue sections from the stored samples described above. For RNA isolation, 1 cm tissue sections were homogenized in TRIzol reagent (Life Technologies, Carlsbad, CA) and the RNA was pelleted. Purification was done using the RNeasy Mini Kit (Qiagen, Hilden, Germany) according to the manufacturer's instructions. A nanodrop instrument (ThermoFisher, Waltham, MA) and Agilent Bioanalyzer (Agilent, Santa Clara, CA) were used to evaluate the concentration and purity of the RNA samples, respectively. The RT^2^ first strand kit (Qiagen) was used to synthesize cDNA from the purified RNA. Gene expression levels were measured using a custom assembled RT^2^ Profiler PCR Assay (Qiagen). Forty select genes spanning multiple aspects of the innate and adaptive immune system and one housekeeping gene (β-actin) on our custom qPCR panel are listed in Table 1. qPCR was performed with a Roche LightCycler 480 (Roche, Basel, Switzerland). The ΔΔCt was calculated and relative expression calculated as 2^−Δ*ΔCt*^ (41).

**Table 1:** Gene abbreviations for the qPCR assays in this study.

**Abbreviation**	**Full Gene Name**
Ccl2	Chemokine (C-C motif) ligand 2
Ccl3	Chemokine (C-C motif) ligand 3
Ccl4	Chemokine (C-C motif) ligand 4
Cx3cl1	Chemokine (C-X3-C motif) ligand 1
Cxcl1	Chemokine (C-X-C motif) ligand 1
Cxcl2	Chemokine (C-X-C motif) ligand 2
Ccl24	Chemokine (C-C motif) ligand 24
Arg1	Arginase 1
Chia1	Chitinase, acidic 1
Chi3l1	Chitinase-3-like protein 1
Chi3l3	Chitinase-3-like protein 3 (YM1)
Mcpt1	Mast cell protease 1
Mcpt4	Mast cell protease 4
Mcpt6	Mast cell protease 6 (Tpsb2)
Gcsf	Granulocyte colony-stimulating factor
Gmcsf	Granulocyte-macrophage colony-stimulating factor
Nos2	Nitric oxide synthase 2
Il17a	Interleukin 17A
Il17f	Interleukin 17F
Il22	Interleukin 22
Il23a	Interleukin 23A
Il6	Interleukin 6
Cxcl9	Chemokine (C-X-C motif) ligand 9
Cxcl10	Chemokine (C-X-C motif) ligand 10
Il12a	Interleukin 12A
Tnf	Tumor necrosis factor
Ifng	Interferon γ
Il18	Interleukin 18
Il1b	Interleukin 1β
Il36g	Interleukin 36γ (Il1f9)
Il25	Interleukin 25
Ccl11	Chemokine (C-C motif) ligand 11
Il33	Interleukin 33
Il4	Interleukin 4
Il5	Interleukin 5
Il13	Interleukin 13
Il9	Interleukin 9
Tslp	Thymic stromal lymphopoietin
Il10	Interleukin 10
Tgfb1	Transforming growth factor β1
Actb	β-actin

### 16s rRNA Sequencing and Microbiome Analysis

Procedures were performed as previously described in detail (42). Briefly, genomic DNA was extracted from mouse tissue (Qiagen DNeasy Blood & Tissue kit) and homogenized in PowerBead Tubes, Garnet 0.70 mm (Qiagen) using a modified protocol previously demonstrated to isolate bacterial DNA. The V4 region of the 16s rRNA gene was amplified using published primers and sequencing was performed using the Illumina MiSeq platform (San Diego, CA), using a MiSeq Reagent Kit V2 (500 cycles). Accuprime High Fidelity Taq was used in place of Accuprime Pfx SuperMix. Primary PCR cycling conditions were 95°C for 2 min, followed by 20 cycles of touchdown PCR (95°C 20 s, 60°C 20 s and decreasing 0.3° each cycle, 72°C 5 min), then 20 cycles of standard PCR (95°C for 20 s, 55°C for 15 s, and 72°C for 5 min), and finished with 72°C for 10 min. Sequence data were processed and analyzed using the software mothur v.1.35.1 according to the Standard Operating Procedure for MiSeq sequence data using a minimum sequence length of 250 base pairs. For each experiment and sequencing run, a shared community file and a phylotyped (genus-level grouping) file were generated using operational taxonomic units (OTUs) binned at 97% identity generated using the dist.seqs, cluster, make.shared and classify.otu commands in mothur. OTU numbers were arbitrarily assigned in the binning process and are referred to throughout the manuscript in association with their most specified level of taxonomy. Classification of OTUs was carried out using the mothur implementation of the Ribosomal Database Project (RDP) Classifier and the RDP taxonomy training set 14 (Trainset14_032015.rdp), available on the mothur website. Sequences used in the 16s rRNA analyses are available via the NCBI Sequence Read Archive (accession number PRJNA745350).

### Statistical Analysis

GraphPad Prism 8 was used in performing significance testing. Outlier datapoints in an assay were identified and removed using Mean Absolute Deviation. Statistical significance for temperature and serological analyses comparing the OVA and untreated groups was determined using the Student's *t*-test with a Bonferroni correction for multiple comparisons. Unpaired two-tailed *t*-tests were used to identify statistically significant differences in gene expression between untreated and treated mice. For all other analyses, statistically significant changes were identified using a One-Way Analysis of Variance ANOVA with Tukey's *post-hoc* test for multiple comparisons. Significance was set at *p* < 0.05 in all analyses. We performed microbial ecology analysis using the *vegan* package 2.2-1 and *mvabund* in R. Ordinations were generated using Principal Component Analysis on Hellinger-transformed normalized OTU tables generated using Euclidean distances. We determined significance of differences in community composition using PERMANOVA (*adonis*) with 10,000 permutations using Euclidean distances.

## Results

For our initial experiments, Balb/cJ mice were sensitized systemically by intraperitoneal injection of chicken egg allergen (ovalbumin, OVA) mixed with the adjuvant alum and then continued gastrointestinal sensitization three times/week by oral gavage with OVA for 2 weeks, as previously reported (29). All measurements were taken after a final seventh oral gavage challenge 3 days later (Figure 1A). Initially, two groups of mice were tested: mice with a conventional microbiome (Conv) and mice with a *C. albicans*-colonized dysbiotic microbiome (Ca/amox). For this latter group, mice received seven days of a broad-spectrum antibiotic (amoxicillin) in their drinking water and a single oral gavage of *C. albicans* (strain CHN1) midway through antibiotic treatment. During amoxicillin treatment, the total bacterial load in the intestinal tract decreased by 10,000-fold and eliminated the colonization resistance to *C. albicans* intestinal colonization. Mice were removed from antibiotic treatment 1 week prior to initiating systemic priming with OVA and alum (Figure 1A), which allowed the total levels of the bacterial microbiome to return to pre-antibiotic levels within the first 24-48 h. However, the bacterial composition of the intestinal tract remained altered in Ca/amox mice for the duration of the experiment (Figure 1B), similar to previous reports with cefoperazone, another β-lactam antibiotic (34, 35, 38). Conventional mice (Conv) remained on standard drinking water during this time period. On the day of challenge (seventh gavage, day 42), mice were monitored for hypothermia and clinical signs of anaphylaxis. Then, blood and tissue samples (jejunum, Ileum, cecum, colon) were collected for serology, host gene expression analyses, *C. albicans* CFU levels, culture-independent 16S rRNA gene-based microbiome analyses and histology.

**Figure 2:**
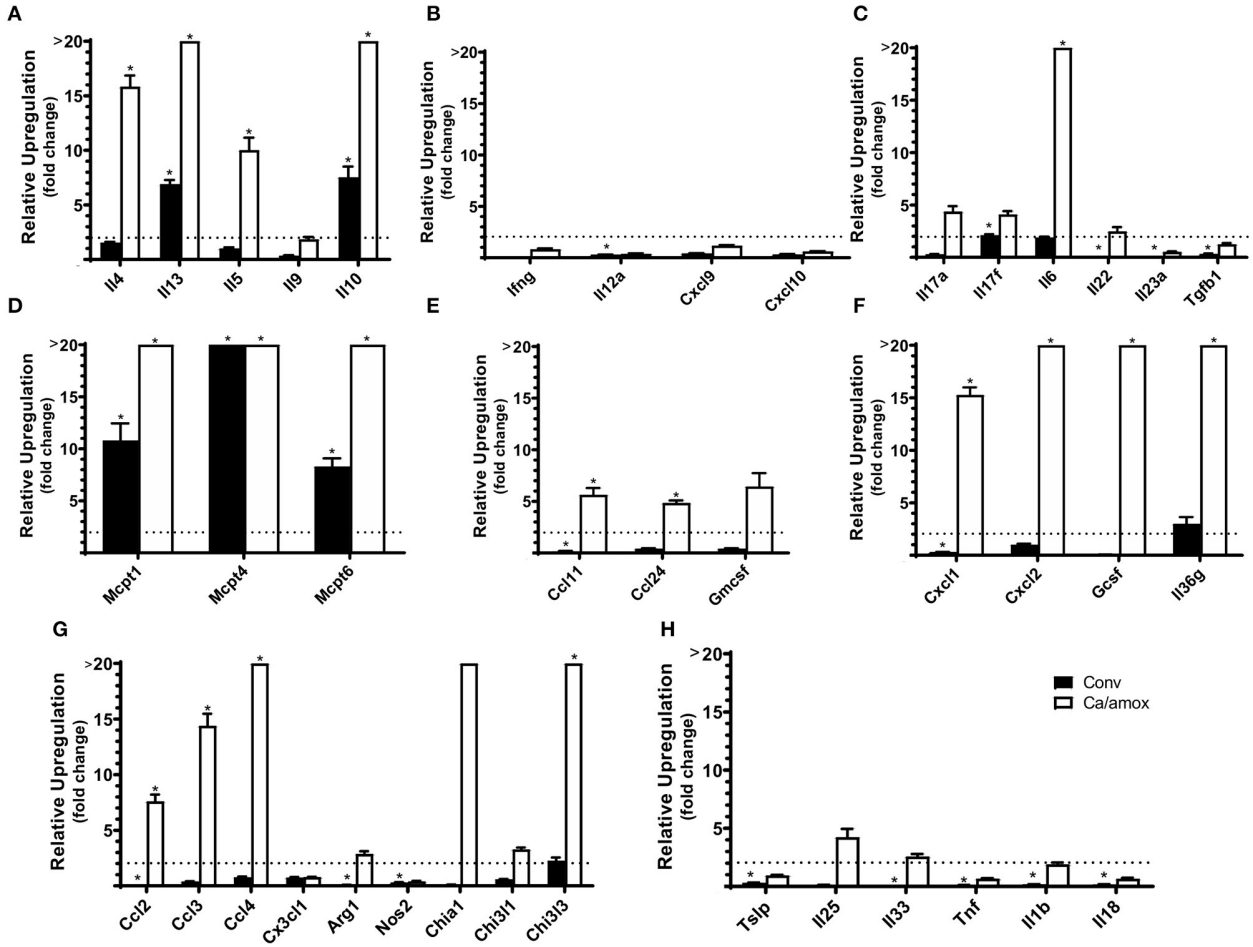
Cytokine and chemokine gene induction in the ileum of Conv and Ca/amox Balb/cJ mice after food allergen (OVA) sensitization and challenge (D42) **(A–H)**. Data shown is relative expression compared to naive strain-matched mice. Mice were treated as outlined in Figure 1A. The dotted line indicates a two-fold upregulation from baseline expression levels. Gene expression was measured by qPCR. Statistical significance (*P < 0.05) signifies significant upregulation in the treated compared to the naïve group for each gene (treated, *n* = 9-10, naïve, *n* = 5). Data are plotted as the mean ± SEM. Detailed description of analyses and statistical tests used can be found in the Materials and Methods. Definitions of gene abbreviations can be found in Table 1.

**Figure 3:**
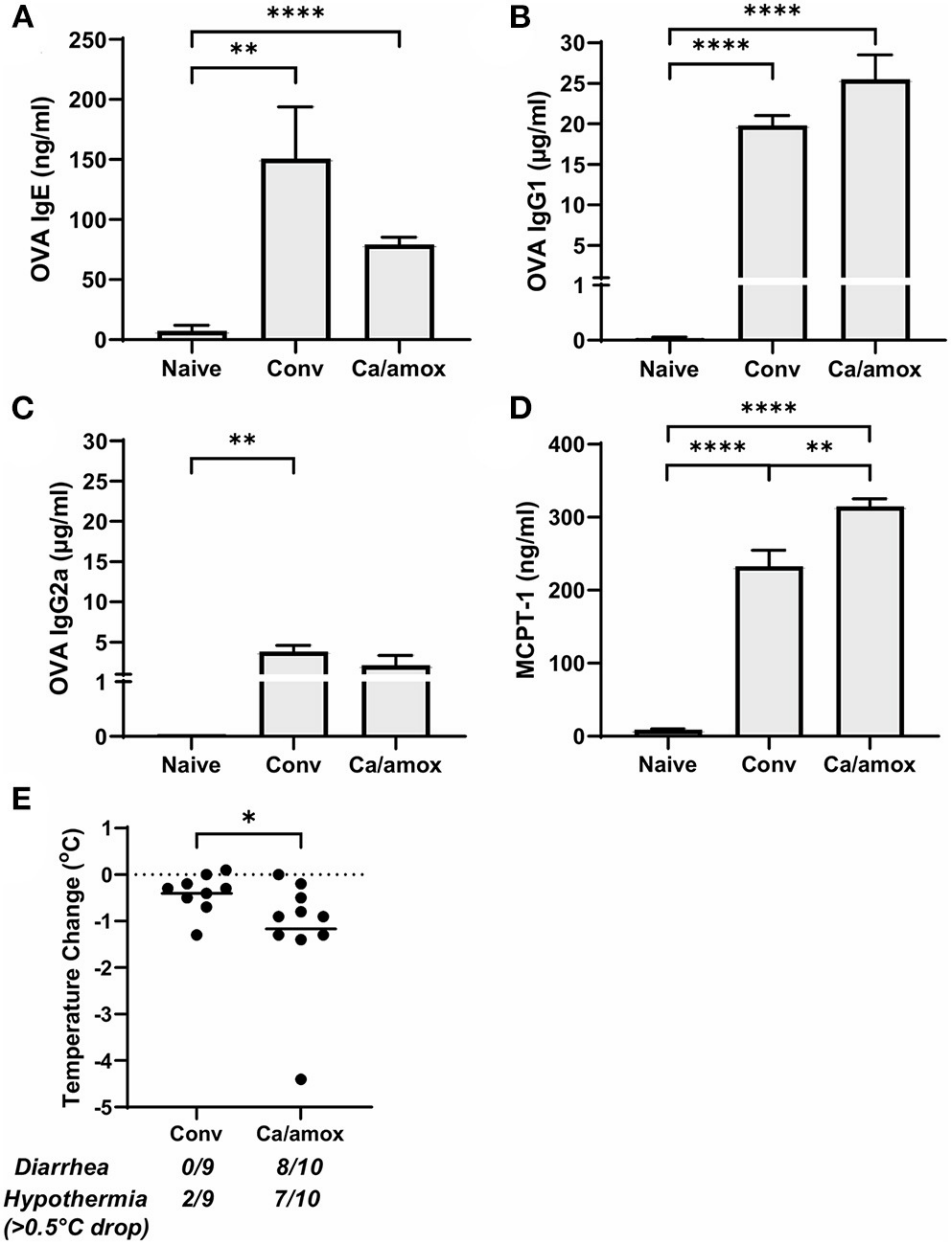
Systemic anaphylaxis indicators measured post-oral antigen exposure in conventional (Conv) and microbiota disrupted (Ca/amox) Balb/cJ mice. Serum antibody and anaphylaxis mediators were collected after challenge seven on D42 and **(A)** OVA-specific IgE **(B)** OVA-specific IgG1, **(C)** OVA-specific IgG2a, and **(D)** MCPT-1 were measured by ELISA. **(E)** The average body temperature drop between 15 and 90 min and the incidence of diarrhea were taken immediately after OVA challenge seven on D42. Shown below the average temperature drop is the number of mice with diarrhea or hypothermia defined as >0.5°C drop out of the total number per group. A single dot represents one mouse and a bar represents the mean ± SEM for the corresponding group: *n* = 9 to 10 mice. *P*-values were determined with a Student's *t-*test. Statistical significance is *P < 0.05, **P < 0.01, and **** *P* < 0.0001.

Ca/amox mice displayed consistent low-level colonization by *C. albicans* (~10^6^ CFU/g) and a bacterial microbiome profile that was distinct from that of Conv mice (Figures 1B,C). The dysbiotic Ca/amox mice had significantly elevated expression levels of the Type 2 cytokines (IL-4, IL-13, IL-5, IL-10), mast cell proteases (Mcpt1, Mcpt4, Mcpt6), chemokines (Ccl2, Ccl3, Ccl4, Ccl11, Ccl24, Cxcl1, Cxcl2), chitinase Ym1/Chi3l3, G-CSF and IL-6 in the ileum (Figure 2). Furthermore, there was a lack of induction of Type 1 and Type 3 (11) cytokines and responses. Similar results were also observed for the colon (Supplementary Figures 2, 3), as well as the duodenum and cecum (data not shown). Overall, the expression of these mucosal markers of allergic disease were much higher in Ca/amox mice than in Conv mice. In contrast, serum levels of OVA-specific IgE were lower in Ca/amox mice, however, serum levels were higher for OVA-specific IgG1 and MCPT-1 (Figures 3A-D). Moreover, clinical indicators of food-allergy induced anaphylaxis, such as hypothermia and diarrhea, were much more pronounced in Ca/amox mice (Figure 3E and Supplementary Figures 1A, B). Thus, intestinal dysbiosis generated by short duration amoxicillin pre-treatment and *C. albicans* colonization can markedly augment food allergen-induced mucosal Type 2 immunity and anaphylaxis.

**Figure 4:**
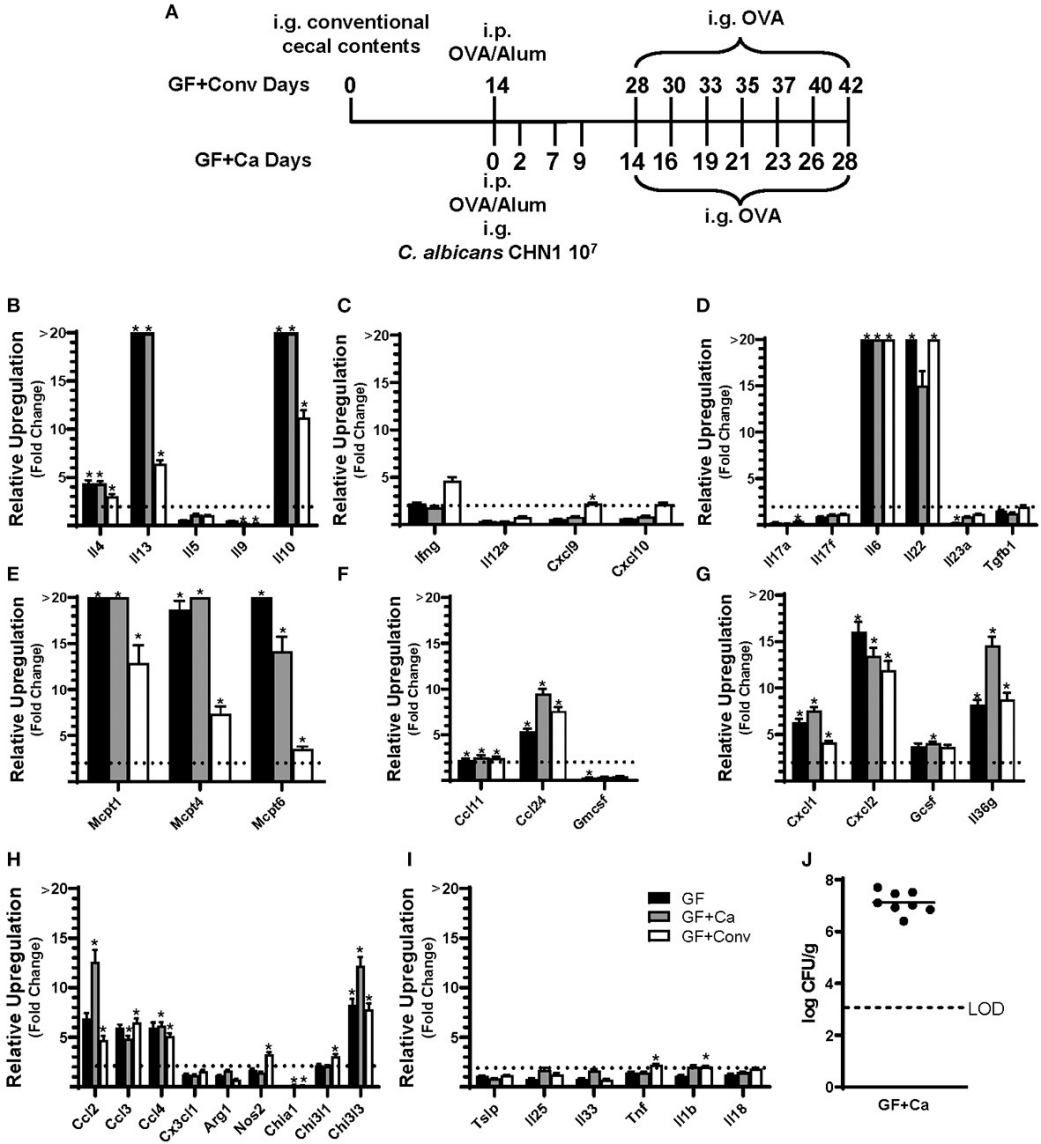
Experimental model and relative expression of cytokine genes in the ileum after OVA sensitization and challenge (D28 GF and GF + Ca or D42 GF + Conv) of three different groups of Balb/cJ mice: germ free (GF), mice monocolonized with *Candida albicans* CHN1 (GF + Ca), and mice conventionalized with cecal contents from Conv donor mice (GF+Conv). **(A)** The same model as described in Figure 1A was used in GF mice, GF + Conv, and GF + Ca. GF + Conv mice were orally gavaged with conventional cecal contents from Conv mice 2 weeks prior to food allergy sensitization. Fecal samples were collected from GF + Ca mice at days 2, 7, 9, 14, 19, 23, and 28. **(B-I)** Mice were treated as outlined in panel A where GF mice were treated like the GF + Ca except the i.g. of *C. albicans* CHN1. Gene expression was measured by qPCR and is shown as relative expression compared to naïve strain-matched mice. The dotted line indicates a two-fold upregulation from baseline expression levels. Statistical significance (*P < 0.05) signifies significant upregulation in the treated compared to the naïve group for each gene (treated, *n* = 7-10, naïve, *n* = 5). Data are plotted as the mean ± SEM. **(J)** CFU of *C. albicans* from the cecal contents. Detailed description of analyses and statistical tests used can be found in the Materials and Methods. LOD is the limit of detection. Definitions of gene abbreviations can be found in Table 1.

**Figure 5:**
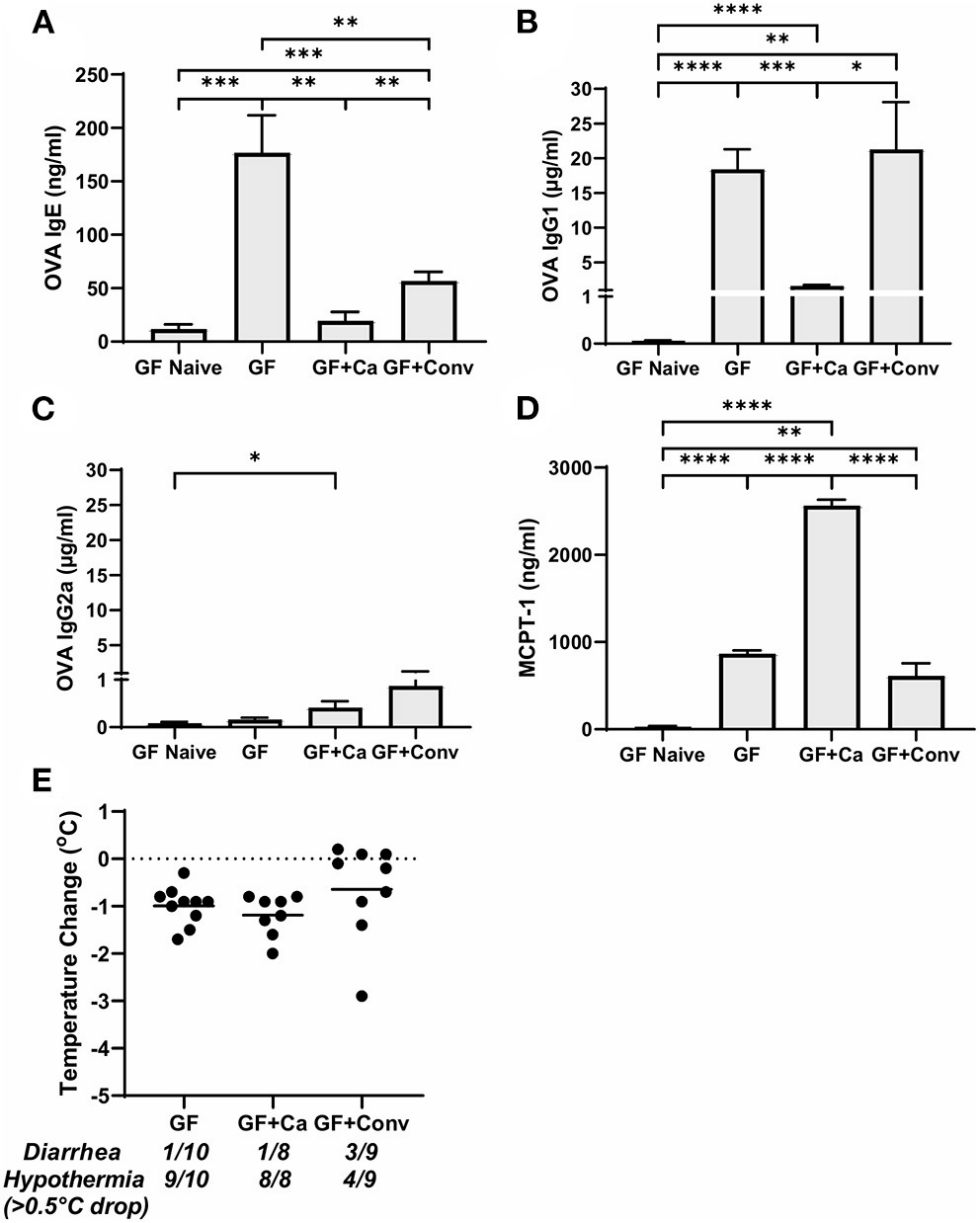
Systemic anaphylaxis markers post-oral antigen exposure in germ-free (GF), CHN1 monocolonized (GF + Ca), and conventionalized (GF + Conv) Balb/cJ mice. Measurements for serum **(A)** OVA-specific IgE, **(B)** OVA-specific IgG1, **(C)** OVA-specific IgG2a, **(D)** MCPT-1, and **(E)** body temperature (0-1.5 h) were collected for the three groups of mice after challenge seven on day 28 (GF and GF + Ca) or day 42 (GF + Conv). The plot shows average body temperature drop between 15 and 90 min, total number of mice with an incidence of diarrhea, and number of mice with hypothermia defined as >0.5°C drop per group. Each dot represents a single mouse, and the bars show mean ± SEM; *n* = 8 to 10 mice. *P*-values were determined with a Student's *t-*test. Statistical significance is *P < 0.05, **P < 0.01, ***P < 0.001, and ****P < 0.0001.

Since changes in the bacterial microbiome were associated with augmentation of the allergic response to a food allergen, our next objective was to determine if the absence of a microbiome would lead to enhanced mucosal Type 2 immunity. Germ-free (GF) Balb/c mice were sensitized systemically and mucosally to OVA as described for the previous experiments and then challenged by oral gavage of OVA (Figures 1A, 4A). Similar to Ca/amox mice, GF mice had significantly elevated expression levels of Type 2 cytokines, mast cell proteases, chemokines, chitinases, and IL-6 in the ileum and lacked induction of Type 1 and Type 3 responses (Figure 4). Similar results were observed for the colon (Supplementary Figures 4, 5). The relative upregulation of these mucosal markers of allergic disease were also markedly higher in GF mice compared to Conv mice (Figures 2, 4). GF mice had elevated levels of OVA-specific IgE, OVA-specific IgG1 and MCPT-1 that were comparable to that observed in Conv mice (Figures 3A-D, 5A-D). Hypothermia (as a result of food-allergy induced anaphylaxis) in GF mice was similar to that observed in Ca/amox mice (and greater than in Conv mice), although the incidence of diarrhea was lower (Figures 3E, 5E and Supplementary Figure 1C). There were some differences in the responses between GF and dysbiotic mice (e.g., lower relative upregulation of cytokines such as IL-4 and IL-5, lack of acidic mammalian chitinase (AMCase; Chia1) induction, enhanced MCPT-1 levels in the blood and lower incidence of diarrhea). However, a complete lack of a microbiome in GF mice markedly augmented food allergen-induced mucosal Type 2 immunity and anaphylaxis.

We next used this germ-free mouse model to identify the relative contribution of *C. albicans* to the dysregulation of the mucosal immune response. Germ-free Balb/c mice were colonized with *C. albicans* strain CHN1 (GF + Ca) at the onset of food allergy sensitization (Figure 4A). This resulted in *C. albicans* levels that were comparable to that observed in the dysbiotic Ca/amox mice (Figure 4I). These mono-colonized mice had nearly identical elevated Type 2 (and lack of Type 1 and Type 3) mucosal immune responses as those in GF mice (Figure 4 and Supplementary Figures 4, 5). There were some serologic differences, including higher levels of MCPT-1 and lower levels of OVA-specific IgE and IgG1, but the GF + Ca mice developed similar anaphylactic responses as those observed in GF mice (Figure 5). Overall, the continued enhanced mucosal Type 2 responses were consistent with intestinal *C. albicans* colonization supporting the development of mucosal Type 2 immune responses.

We subsequently studied the effect of colonizing germ-free Balb/c mice with the intestinal microbiome from conventional Balb/cJ mice on the development of mucosal Type 2 immunity and anaphylaxis. Germ-free Balb/c mice were given an oral gavage of the cecal microbiome from a naive conventional Balb/cJ mouse and then sensitized and challenged in the food allergy model, beginning 14 days after conventionalization. As we have previously published, this protocol is sufficient to establish a fully functional microbiome between days 7-21 (43). In contrast to *C. albicans* mono-colonization (GF + Ca), and consistent with Conv mice, conventionalized germ-free mice (GF + Conv) had lower induction of Type 2 cytokines and mast cell proteases than GF mice (Figure 4). Expression of chemokines and chitinases did not significantly change compared to GF mice, but the overall up regulation of these genes was lower than what was observed in dysbiotic (Ca/amox) mice (Figures 2, 4). Levels of OVA-specific IgE were lower but the serologic levels of OVA-specific IgG1 and MCPT-1 were comparable to that observed in GF mice (Figures 5A-D). However, GF + Conv mice had a markedly reduced hypothermia response, with only 4/9 mice responding compared to 9/10 GF mice responding (Figure 5E and Supplementary Figures 1C-E). These data support the concept that the bacterial microbiome contains species or communities that protect against induction of food allergen-induced intestinal Type 2 immunity and anaphylaxis in genetically susceptible mice and suggest that GF mice and dysbiotic mice (Ca/amox) lack these.

Our data led us to consider another possible variable in our *in vivo* experiments: environment. All of our initial experiments were tightly controlled for mouse strain (Balb/cJ), vendor (Jackson Labs), shipments, entry room in the animal facility, sex (female), protocol, parasite status (negative), source of reagents, lack of endotoxin contamination in the food allergen, type of mouse chow, source of water, type of caging (filter top), cage change protocols, bedding and number of mice per cage. However, if mice were treated with antibiotics and colonized with *C. albicans*, they were moved from biosafety level 1 conditions to a different barrier room nearby in the animal facility for the remainder of the experiment because they now harbored *C. albicans*, a biosafety level 2 microbe. Thus, we wondered whether some aspect of the new room itself could affect the robustness of this murine food allergy model. For the next set of experiments, mice were moved from our standard specific pathogen free BSL1 room into the BSL2 barrier room at our animal facility. Two weeks later, mice were sensitized systemically and mucosally to OVA, followed by a final oral gavage challenge with OVA. All mice in these experiments were culture negative for *C. albicans* and other yeast (as defined by growth on SDA plates with cefoperazone).

**Figure 6:**
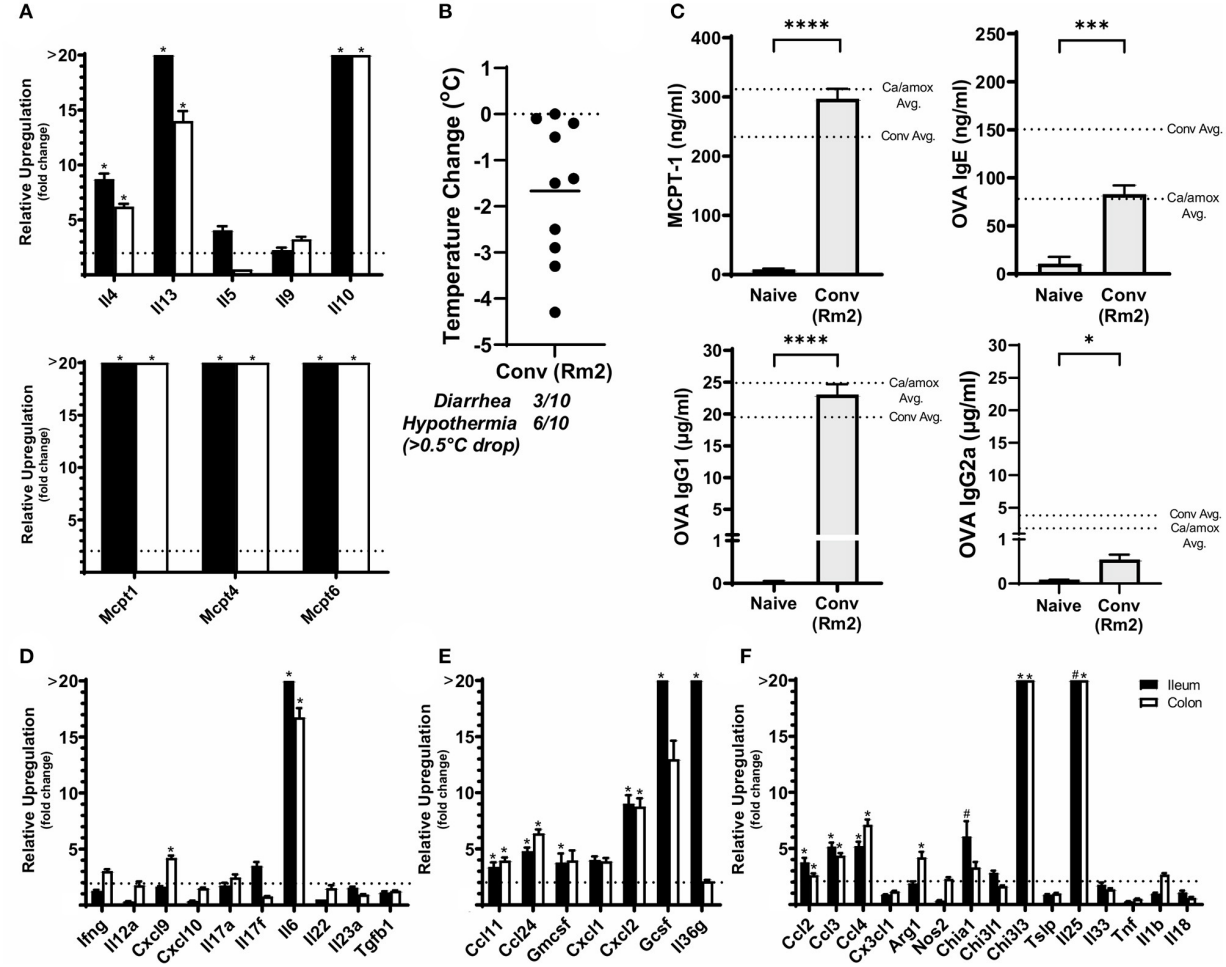
Anaphylaxis measurements and relative cytokine gene expression after the final challenge in food allergic Balb/cJ mice tested for environmental impact on the microbiome (Conv Rm2). **(A,D–F)** Relative expression of cytokine genes in the ileum and colon of Conv (Rm2) mice post final challenge. **(B)** Average body temperature drop from 15 to 90 min in sensitized/challenged mice, total number of mice with an incidence of diarrhea, and number of mice with hypothermia defined as >0.5°C drop per group. Body temperature was recorded after the final oral challenge via rectal probe. **(C)** Average serum levels of MCPT-1, OVA-specific IgE, IgG1 and IgG2a for Conv (Rm2) mice compared with averages of Conv and Ca/amox groups (Figure 3). Mice were treated as outlined in Figure 1A starting at D13. Gene expression was measured by qPCR and is shown as relative expression compared to naïve strain-matched mice. The dotted line indicates a two-fold upregulation from baseline expression levels. Statistical significance (*P < 0.05) signifies significant upregulation in the treated compared to the naïve group for each gene (treated, *n* = 10, naïve, *n* = 5). A bar notated with a # indicates an average upregulation > 5 across the group, but *P* > 0.05 due to high variance in the overall dataset. Data are plotted with the mean ± SEM. Detailed description of analyses and statistical tests used can be found in the Materials and Methods. Statistical significance is ^*^P < 0.05, ***P < 0.001, and ****P < 0.0001. Definitions of gene abbreviations can be found in Table 1.

While not completely recapitulating the robust mucosal Type 2 immune response and systemic anaphylaxis observed in Ca/amox mice, these mice [Conv (Rm2)] nonetheless had a higher incidence of hypothermia and diarrhea during food allergen challenge than Conv mice (Figure 6 and Supplementary Figure 1F). Conv (Rm2) mice also had markedly higher expression levels of Type 2 cytokines, mast cell proteases, chemokines, chitinases, G-CSF and IL-6 in the ileum and colon than Conv mice (Figure 6). The most notable difference in Conv (Rm2) mice vs. Ca/amox mice was the high expression of IL-25 in the ileum and colon. It is also critically important to note that Conv (Rm2) mice did not have upregulation of any genes associated with Type 1 or Type 3 responses, thus making it unlikely that they acquired an intestinal pathogen while housed in filtertop cages in this new room (Figure 6). We have since studied two additional sets of animal rooms in our facility and also found similar room-based effects in altering the magnitude of the Type 2 response and the rate of developing an anaphylactic reaction (data not shown).

**Figure 7:**
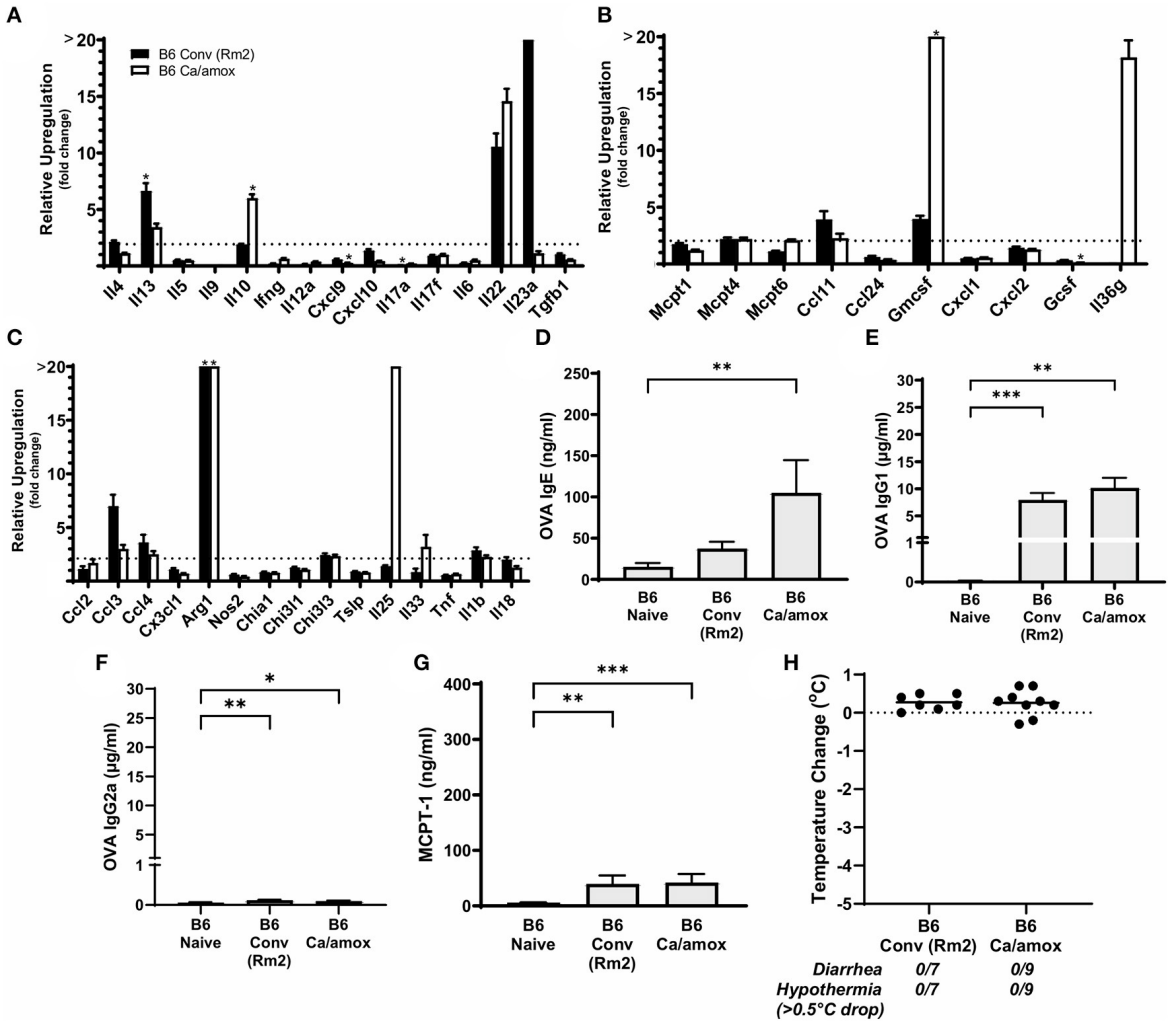
Systemic anaphylaxis markers and cytokine gene induction for C57BL/6 mice tested for environmental impact on the microbiome (B6 Conv Rm2) and microbiota disruption (B6 Ca/amox) after oral antigen exposure. Mice were treated as outlined in Figure 1A. **(A-C)** Cytokine expression shown is relative expression compared to naïve strain-matched mice. The dotted line indicates a two-fold upregulation from baseline expression levels. Gene expression was measured by qPCR. **(D-G)** The immunoglobulin and protease serum levels for **(D)** OVA-specific IgE, **(E)** OVA-specific IgG1, **(F)** OVA-specific IgG2a, and **(G)** MCPT-1. **(H)** Temperature drop was measured *via* rectal probe and the average maximum decrease from baseline was plotted. A single dot represents one mouse, and the bar is the group mean ± SEM. Refer to the materials and methods for a detailed description of analyses and statistical tests performed. Statistical significance: **(A–C)** (*P < 0.05) signifies significant upregulation in the treated compared to the untreated group for each gene (treated, *n* = 7-9, untreated, *n* = 5). *P*-values for **(D–G)** were determined with a Student's *t*-test. Statistical significance is *P < 0.05, **P < 0.01, and ***P < 0.001. Definitions of gene abbreviations can be found in Table 1.

Since our data supported a role for the microbiome and environment in modulating mucosal Type 2 immunity and anaphylaxis in genetically susceptible mice (Balb/cJ), we also tested whether these variables could promote the development of the response in genetically resistant mice (C57BL/6J mice). Using the identical protocol as described previously in this report (Figure 1A), we were unable to induce mucosal Type 2 immunity or anaphylaxis in C57BL/6J mice as a result of dysbiosis (Ca/amox) or housing (Rm2) (Figure 7). We were able to induce some low-level changes in systemic Type 2 immunity, as indicated by elevated OVA-specific IgE in dysbiotic C57BL/6J mice, but these were insufficient to elicit an anaphylactic reaction in the mice (Figure 7 and Supplementary Figure 6).

**Figure 8:**
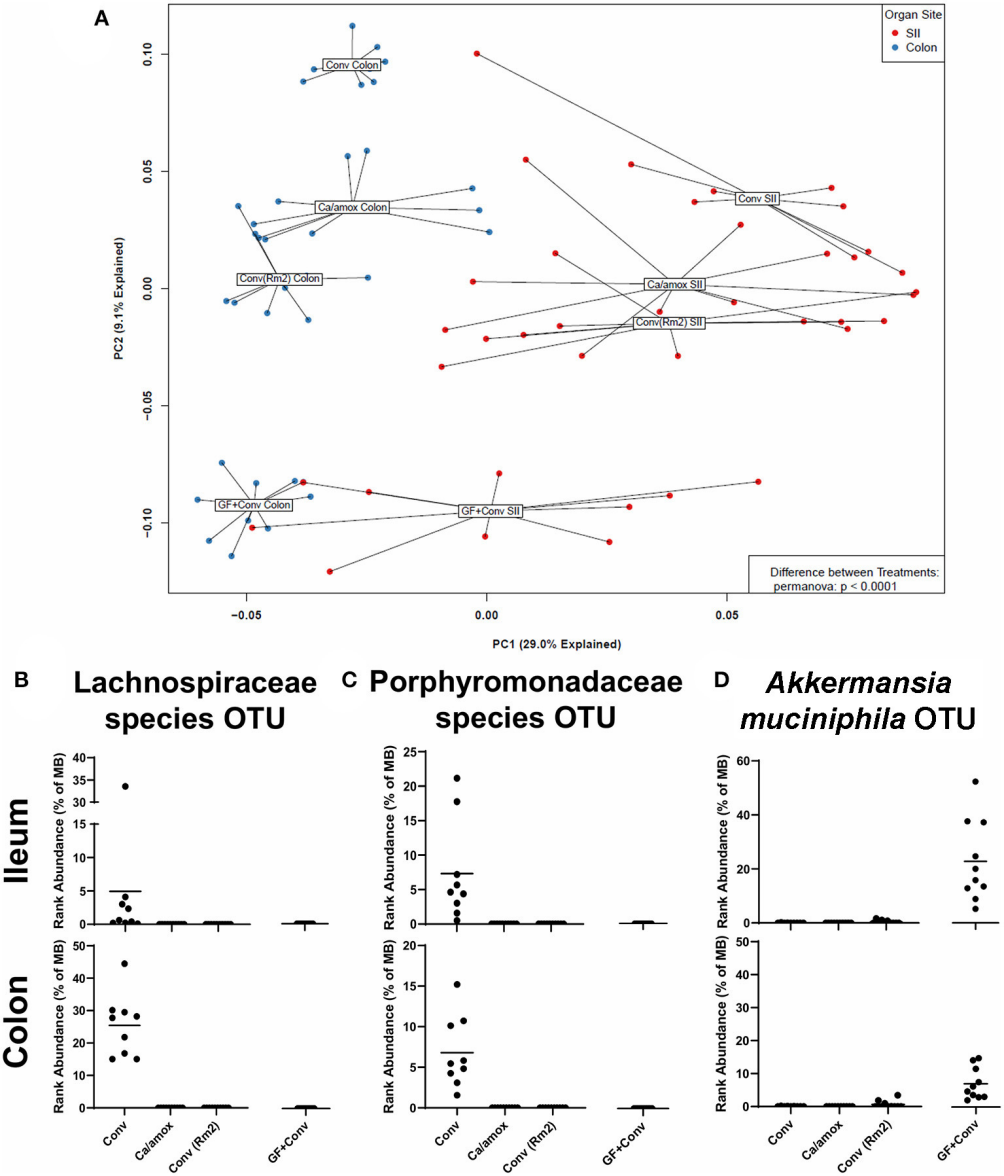
Disparate microbiome composition in the small intestine ileum (SII) and colon of Conv, Ca/amox, Conv (Rm2), and GF + Conv mice after oral antigen exposure. The four experimental groups were treated according to Figures 1A, 4A. Tissue snips of the ileum and colon were collected post-oral antigen exposure on D28 (Conv Rm2 and Conv) or D42 (GF + Conv and Ca/amox), and DNA of the mucosally-associated bacteria was extracted for 16s rRNA sequencing. **(A)** PCA of distinct bacterial communities in the ileum and colon of the four groups after sensitization and challenge to OVA. **(B-D)** Percent rank abundance of three dominant species within the microbiome of the Conv or GF + Conv mice. Each dot represents a single mouse, and the *p*-values were determined with a permanova. See 16s rRNA Sequencing and Microbiome Analysis section for detailed methods of microbiome data processing.

We next set out to determine if specific bacterial species or communities in the ileum and colon were associated with high vs. low responses in genetically susceptible Balb/cJ mice in the experiments reported here. Bacterial microbiome analyses were carried out using culture-independent 16S rRNA gene amplicon sequencing from metagenomic DNA isolated from the ileum and colon of individual mice within each group. OTUs were created by binning sequences at 97% identity. The data from these analyses are shown as a principal component analysis in Figure 8A. As previously discussed for Figure 1B, the Ca/amox mice had a microbiome whose community structure was significantly different than that of Conv mice. Moreover, the microbiome of the Conv (Rm2) mice was also significantly different than the microbiome of Conv mice and was more similar in structure to that of the Ca/amox mice. The microbiome of the GF+Conv mice was distinct from that of the other groups of mice.

In the microbiome analysis, changes in the community structure were strongly associated with the development of anaphylactic hypothermia and intestinal diarrhea in which two bacterial OTU were identified in Balb/cJ. One was an OTU that could be assigned to the family *Lachnospiraceae* and the other was an OTU that could be assigned to the family *Porphyromonadaceae*. We subsequently performed shotgun metagenomic sequencing and analysis on one of the samples containing high levels of the *Lachnospiraceae* OTU. We were able to collect enough reads of sufficient length to provide a tentative identification as a *Lachnoclostridium* species similar to *Lachnoclostridium* YL32. As illustrated in Figures 8B,C, these two bacterial OTUs were present in the group of genetically susceptible mice that did not develop robust mucosal Th2 immunity, diarrhea, or anaphylaxis (Conv) and were absent in the groups of mice that did [Ca/amox and Conv (Rm2)]. Of note, these OTU were not found in the GF +Conv group; however, there was another notable OTU that was found only in this group of mice (which is the primary driver of their distinct PCA clustering). This OTU could be clearly identified as *Akkermansia*, of which only one species has been identified in mice, *Akkermansia muciniphila* (Figure 8D). Altogether, our analyses of the intestinal microbiome indicate that there likely exists a number of distinct bacterial species, whose presence in the GI tract is associated with reduced food allergen mucosal Type 2 immunity and anaphylaxis. Furthermore, the persistence of *C. albicans* in the gut can augment these responses in genetically susceptible mice (Balb/c), but *C. albicans* colonization alone is not sufficient to drive robust mucosal Type 2 responses in genetically resistant mice (C57BL/6J).

## Discussion

In this study, we noted that the intensity of the anaphylactic reactions was most strongly associated with a disrupted microbiome in Balb/c mice that included colonization by *C. albicans*, loss of a specific *Lachnoclostridium* species, development of a highly polarized Type 2 response in the intestinal mucosa and underlying tissue, and activation of mucosal mast cells. Serum levels of allergen-specific IgE were not predictive of the response and a complete absence of a microbiome did not fully recapitulate the response. Conventionalization of germ-free mice resulted in *Akkermansia muciniphila* outgrowth and a higher degree of heterogeneity in the allergic response, consistent with the idea that there exist multiple microbiome-mediated pathways that can modulate mucosal Type 2 immunity. Genetically resistant C57BL/6 mice remained resistant to food allergy induction even under the same dysbiosis-inducing antibiotic regimens. We also observed that different rooms in our vivarium could induce microbiome changes that were associated with higher or lower Type 2 responses and clinical parameters of anaphylaxis. Thus, our data recapitulate the heterogeneity in anaphylactic reactions, ranging from severe to none, seen in patients that have circulating levels of food allergen-reactive IgE and support the concept that alterations in the microbiome can be one factor underlying this heterogeneity.

There was marked gene expression of MCPT-1, −4, and −6 (as well as MCPT-1 in the serum) observed in all Balb/cJ mice, especially when colonized with *C. albicans*. Mast cells are one of the major contributing cell types to the various symptoms of allergic disease and respond to a variety of environmental stimuli. Mast cells are prevalent throughout the body and can be tissue-specific (26, 27). Resident MMCs are typically activated by IL-9 and mediate the release of MCPTs and other effector molecules through antigen-specific IgE bound to its corresponding cell surface receptor, FcεR1 (8, 26–29). How does this gene expression correlate to the levels of mast cells in the tissue? The answer to this question remains to be determined. We have not performed a comprehensive histological or flow cytometric analysis for mast cells in the intestinal tissue across all the groups. However, we have performed chloroacetate esterase staining for mast cells in a small cohort of untreated and sensitized mice for the Conv (Rm2) Balb/cJ group (Supplementary Figure 7), which clearly illustrates a marked expansion of mast cells. A more comprehensive analysis needs to be done in order to draw conclusions about how the levels of mast cells in the tissue correlate with the changes in the transcriptional profile. Alternative activation pathways may also be active as evidenced by increased levels of IgG1, which have been shown to act synergistically with antigen-specific IgE and exacerbate the severity of anaphylaxis (44). Additionally, this is seen regarding the presence of *C. albicans* inducing IL-9 production after phagocytosis by MMCs leading to epithelial damage and inflammation (40). Furthermore, *C. albicans* colonization was not only shown to alter the integrity of the gastrointestinal mucosal barrier promoting OVA leak in a mast cell- dependent manner in Balb/c mice (39), but also prevent the suppression of OVA-specific antibody production leading to an inhibition of humoral tolerance (45). Contrary to our data, one study found that germ-free mice have impaired mast cell functionality and do not develop food allergy. Schwarzer et al. observed lower amounts of CXCL1 and CXCL2, lack of hypothermia, and lower levels of MCPT-1 (46). Taken together, it appears there is strong evidence for the implication of *C. albicans* colonization and for the microbiome affecting mucosal barrier integrity, through influencing mast cell activation.

We saw an association between the presence of specific species of *Lachnospiraceae, Porphyromonadaceae* or *Akkermansia* (likely *muciniphila*) and the development of food allergy in Conv, GF+Conv, and Ca/amox and Conv (Rm2) mice. Dysbiosis of the microbiome has been associated with many disease states from metabolic disease to inflammatory bowel disease, however, there is growing evidence for disease mitigation by particular species of bacteria (47–52). Some species of *Lachnospiraceae* can produce metabolic byproducts such as butyrate, a short chain fatty acid (53). Many studies have found *A. muciniphila* to be correlated with amelioration of metabolic syndrome, which is thought to be related to its metabolism of the mucus lining the GI tract (54, 55). In food allergy, we are only just beginning to investigate the mechanisms of how the microbiome influences disease state. Experiments have recently been reported in which inoculation of Clostridiales species, either as a consortium or as monotherapy with *Subdoligranulum variabile*, or a Bacteroidales consortium could reduce development of food allergy in OVA sensitized mice by acting through MyD88 and RORγt (56). An association between *Subdoligranulum variabile* and *A. muciniphila* has also been reported in a study of overweight/obese individuals (57). Other studies have noted associations between a signature food allergic microbiome and promotion of OVA-specific IgE and anaphylaxis when reconstituted in germ-free mice (58). The authors identified OTU's mapping to *Lachnospiraceae* and *Porphyromonadaceae* in their cohorts of mice resistant to development of food allergies, which is consistent with our findings.

There is a robust Type 2 dominant response in the mucosa, with high levels of mast cell activation, and little to no contribution from Type 1 or Type 3 responses in mice that developed food allergies. IL-17 and Type 3 immunity, which includes Th17 cells (11), have been implicated in the pathology of other allergic diseases such as asthma (59–62). There is a correlation between increased levels of IL-17 expression and more severe disease (60, 61, 63). However, our results reveal a markedly different story in the context of food allergy, where neither Type 3 nor Type 1 responses increased significantly after sensitization and food allergen challenge in either susceptible Balb/c or resistant C57BL/6 mice (Figures 2, 7). These results are consistent with other reports of little expression of IL-17 measured in CD4+ T cells of food-allergic human patients *ex vivo* or *in vitro* after food antigen stimulation (64). This provides additional insights into the mucosal immune response occurring in this murine model of food allergy (28–30).

We observed high expression levels of IL-6 in addition to high numbers of mast cells and Type 2 cytokines in Balb/c but not C57BL/6 mice. IL-6 is a pleiotropic cytokine with both pro- and anti-inflammatory properties and plays a role in T follicular helper (Tfh) cell development (65, 66). Of the many functions of IL-6, some of the most pertinent to this study are those involved in the inflammatory processes (65, 67, 68). IL-6 has been shown to be important in the decision between differentiation and maturation of Th9 and Th17 cells along with helping to expand the Th2 cell population (69). In addition, there is substantial evidence for IL-6 and its interaction with mast cells not only for wound healing but also in enhancing proliferation and functionality (67, 70, 71). Clinical observations in humans have been made correlating IL-6 levels with severity of systemic mastocytosis, asthma and related diseases involving mast cell activity (67, 72–74). Evidence exists showing that proliferation and formation of human mast cells, comprising a highly reactive phenotype, is enhanced by IL-6 expression (67). Thus, the impacts of having increased expression of IL-6 in a food allergic response may affect mast cell numbers and function, which are high in the Balb/c mice. It may also alter levels of some of the other cytokines such as IL-9 due to moderate levels of IL-4 and low levels of TGF-β1 not driving Th9 differentiation as reported in Schutze et al. (69).

In food allergic Balb/c mice, we found a lack of alarmin (IL-33, IL-25, or TSLP) production along the intestinal epithelium. This is surprising because alarmins are primary early response signals released after cellular damage that promote mucosal inflammatory immune responses as a defense against invaders (75–78). In particular, the induction of the Type 2 inflammatory response during asthma pathogenesis has been linked to the presence of alarmins at mucosal surfaces (79, 80). IL-33 is a potent activator of mast cells, yet we have observed mast cell activation without increased expression of IL-33 (81). Our hypothesis is that systemic sensitization with alum did not induce epithelial damage; therefore, expression of IL-25, IL-33 and TSLP was minimal. In contrast, when cholera toxin is used as an adjuvant there is epithelial damage, alarmin production, and antigen leak (82, 83). Our data indicates that intestinal alarmin production may not be absolutely required for a Type 2 response against a food allergen if sensitization has occurred at an extra-intestinal site. It should be noted, however, that the Conv (Rm2) showed relatively high upregulation of IL-25. It is known that IL-25 can be produced by Th2 cells along with ILC2s, macrophages and other monocytes, which may be a reason for upregulation of this cytokine in these mice (84).

IL-10 expression was also significantly increased in Balb/c mice that developed systemic anaphylaxis. Recent evidence has suggested that IL-10, traditionally thought of as an anti-inflammatory cytokine, can help drive priming of mast cells and enhance IgE-mediated activation (27, 85). IL-10 has been shown to be essential to mast cell survival, proliferation, and function (85–87). Thus, these pleiotropic effects exhibited by IL-10 in particular by promoting mast cell expansion and activation is consistent with the high levels seen in the Balb/c mice (Figure 2) (85). Overall, our data suggest that IL-10 may help prime mast cells during IgE-mediated food allergy.

Our study illustrates that the environment can influence GI microbial structure which, together with genetics, can play a fundamental part in susceptibility to the development and manifestation of a food allergy and anaphylaxis. The mice used in this study had no evidence of infection, and other variables were held constant (bedding, food, cage types, number of mice per cage). Our experiments used a model with one IP sensitization prior to oral challenges and we could demonstrate an association between microbiome community structure and robustness of the mucosal Type 2 immune response. Other investigators have used a similar model, but with two IP sensitizations, and have reported strong Type 2 responses to food allergen challenge (14, 28, 88), supporting the possibility that a strong systemic sensitization can modulate the influence of the microbiome or other environmental variables. In addition, a study by Leyva-Castillo and colleagues demonstrated that additional factors such as mechanical injury to an inductive mucosal surface can promote food allergy in Balb/c mice (89). Thus, environmental variables, some of which are captured by the inherent design of an experiment like different rooms in the same institution's animal housing facilities, may impact the mucosal Type 2 response and anaphylaxis in scenarios where the mucosal sensitization is modest. These may include variables such as indigenous phage levels in a room, ambient noise, temperature, bedding microenvironments, etc. Of note, we have since studied two additional sets of animal rooms in our facility and found room-based effects in altering the microbiome and susceptibility to the development and manifestation of food allergy (data not shown), as well as had discussions with other investigators at other institutions who have noted the same phenomena.

## Data Availability Statement

The datasets presented in this study can be found in online repositories. The names of the repository/repositories and accession number(s) can be found at: https://www.ncbi.nlm.nih.gov/, PRJNA745350.

## Ethics Statement

The animal study was reviewed and approved by University of Michigan Institutional Animal Care and Use Committee.

## Author Contributions

KS, NF, and GH conceived, designed, and interpreted the experiments. KS, NF, and RM performed the experiments. KS, NF, CB, and GH analyzed the data. KS and GH wrote the manuscript. NF, CB, and RM reviewed and provided advice on the manuscript prior to submission. All authors contributed to the article and approved the submitted version.

## Funding

This work was supported in part by NIH grant NIAID R01AI138348, the Mary H. Weiser Food Allergy Center (MHWFAC), and the Nina and Jerry D. Luptak Endowment of the MHWFAC. This research was also supported in part through computational resources and services provided by Advanced Research Computing (ARC) and a division of Information and Technology Services (ITS) at the University of Michigan, Ann Arbor.

## Conflict of Interest

The authors declare that the research was conducted in the absence of any commercial or financial relationships that could be construed as a potential conflict of interest.

## Publisher's Note

All claims expressed in this article are solely those of the authors and do not necessarily represent those of their affiliated organizations, or those of the publisher, the editors and the reviewers. Any product that may be evaluated in this article, or claim that may be made by its manufacturer, is not guaranteed or endorsed by the publisher.

## Abbreviations

IL: Interleukin
GF: Germ-free
Ig: Immunoglobulin
OTU: Operational Taxonomic Unit
PCA: Principal Component Analysis
MCPT: Mast cell protease
OVA: ovalbumin
Tfh: T follicular helper cells
Th2, T Helper 2 cells;ILC2: Type 2 innate lymphoid cells
MMCs: mucosal mast cells
i.g.: intragastric
CAE: chloroacetate esterase
ST2: suppression of tumorigenicity.
