# Supplementary material for: Contribution of the Microbiome, Environment, and Genetics to Mucosal Type 2 Immunity and Anaphylaxis in a Murine Food Allergy Model
Source: Front Allergy. 2022 Mar 31;3:851993. doi: 10.3389/falgy.2022.851993 (PMC9234882; doi:10.3389/falgy.2022.851993)
Supplement: Supplementary file 1 [file Data_Sheet_1.PDF]

Supplemental Figures

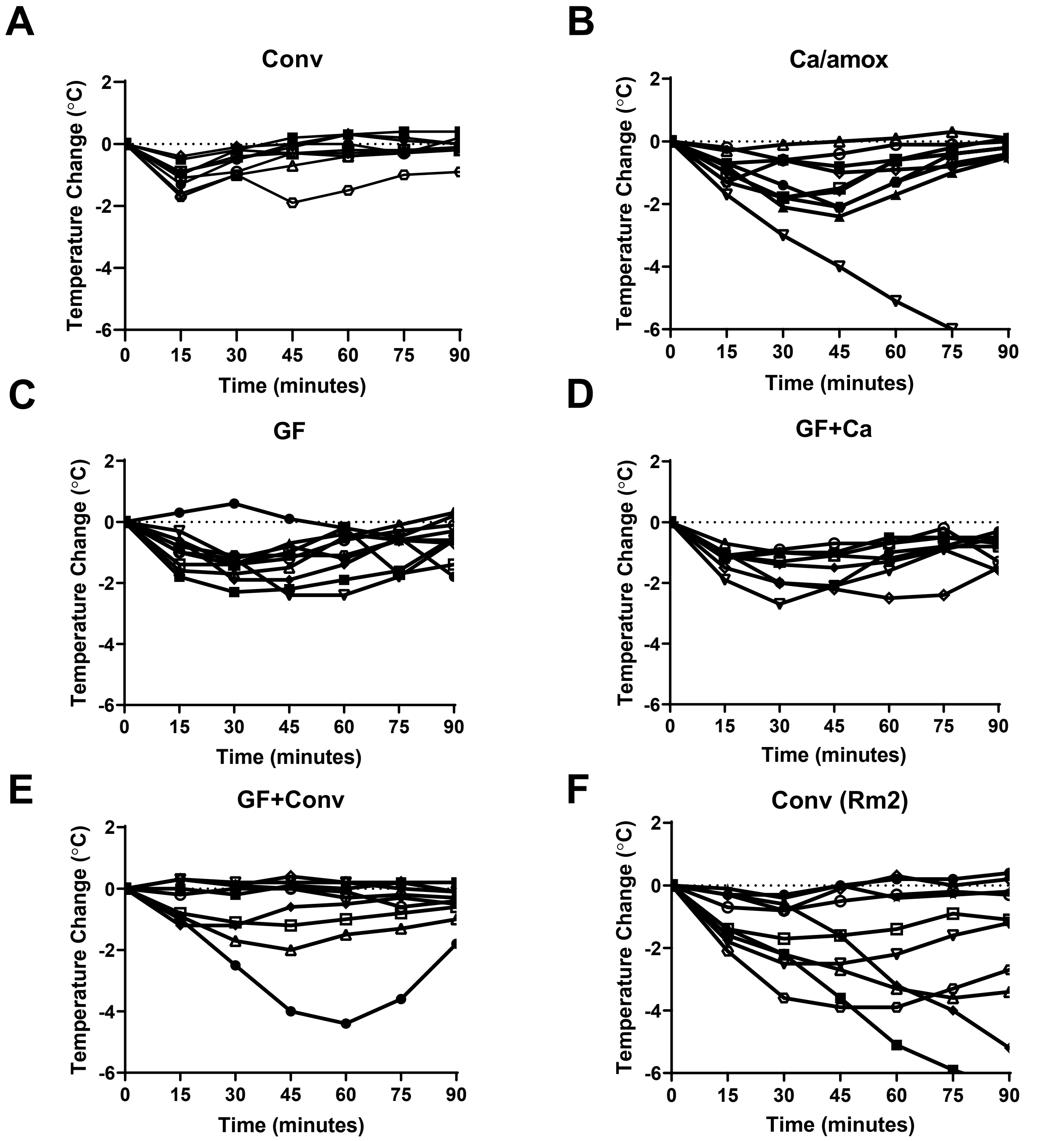

Supplemental Figure 1: Individual body temperature changes of sensitized and challenged food allergic Balb/cJ mice. Body temperatures of **(A)** Conv, **(B)** Ca/amox, **(C)** GF, **(D)** GF+Ca, **(E)** GF+Conv, and **(F)** Conv (Rm2) mice were recorded prior to the final oral challenge and every 15 minutes post-challenge up to 90-minutes via rectal probe (Physitemp Instruments, Clifton, New Jersey). Data are plotted with the mean ± SEM. Detailed description of analyses used can be found in the Materials and Methods.

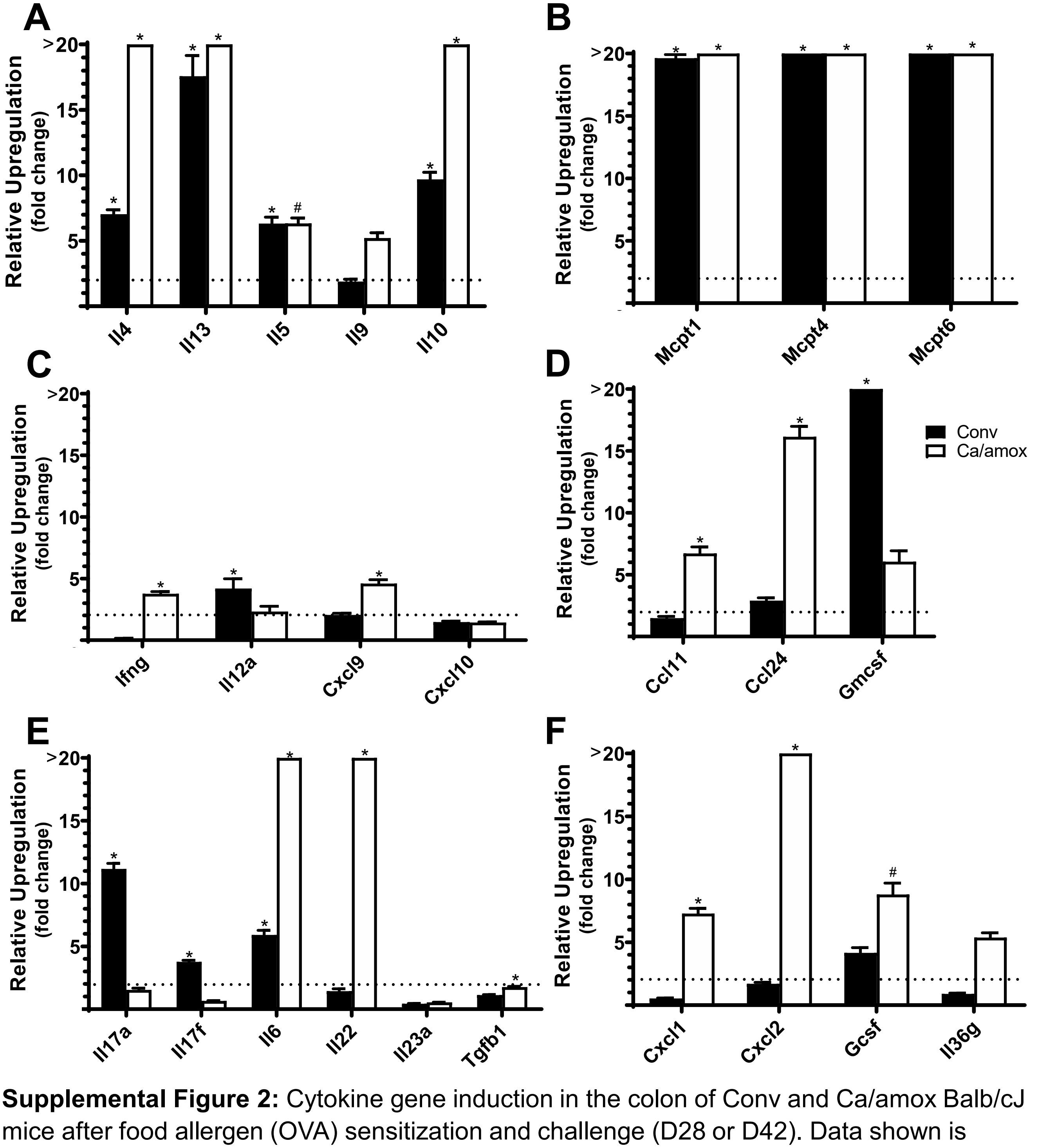

Supplemental Figure 2: Cytokine gene induction in the colon of Conv and Ca/amox Balb/cJ mice after food allergen (OVA) sensitization and challenge (D28 or D42). Data shown is relative expression compared to naïve strain-matched mice. Mice were treated as outlined in Figure 1A. The dotted line indicates a 2-fold upregulation from baseline expression levels. Gene expression was measured by qPCR. Statistical significance (*$P < 0.05$) signifies significant upregulation in the treated compared to the naïve group for each gene (treated, n = 7-10, naive, n = 5). A bar notated with a # indicates an average upregulation greater than 5 across the group, but $P > 0.05$ due to high variance in the overall dataset. Data are plotted as the mean ± SEM. Detailed description of analyses and statistical tests used can be found in the Materials and Methods. Definitions of gene abbreviations can be found in Table 1.

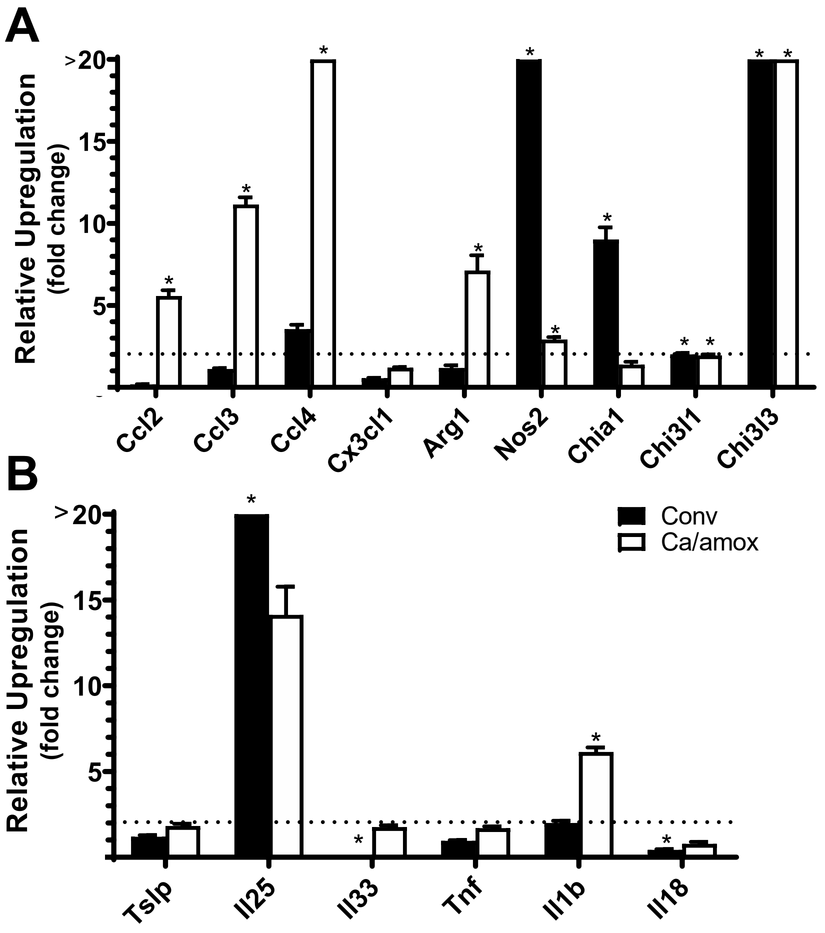

Supplemental Figure 3: Chemokine and cytokine gene expression in the colon of Conv and Ca/amox Balb/cJ mice after OVA sensitization and challenge (D28 or D42). Data shown is relative expression compared to naïve strain-matched mice that were administered treatment as outlined in Figure 1A. The dotted line indicates a 2-fold upregulation from baseline expression levels. Gene expression was measured by qPCR. Statistical significance (**P* < 0.05) signifies significant upregulation in the treated compared to the naïve group for each gene (treated, *n* = 7-10, naïve, *n* = 5). A bar notated with a # indicates an average upregulation greater than 5 across the group, but *P* > 0.05 due to high variance in the overall dataset. Data are plotted as the mean ± SEM. Detailed description of analyses and statistical tests used can be found in the Materials and Methods. Definitions of gene abbreviations can be found in Table 1.

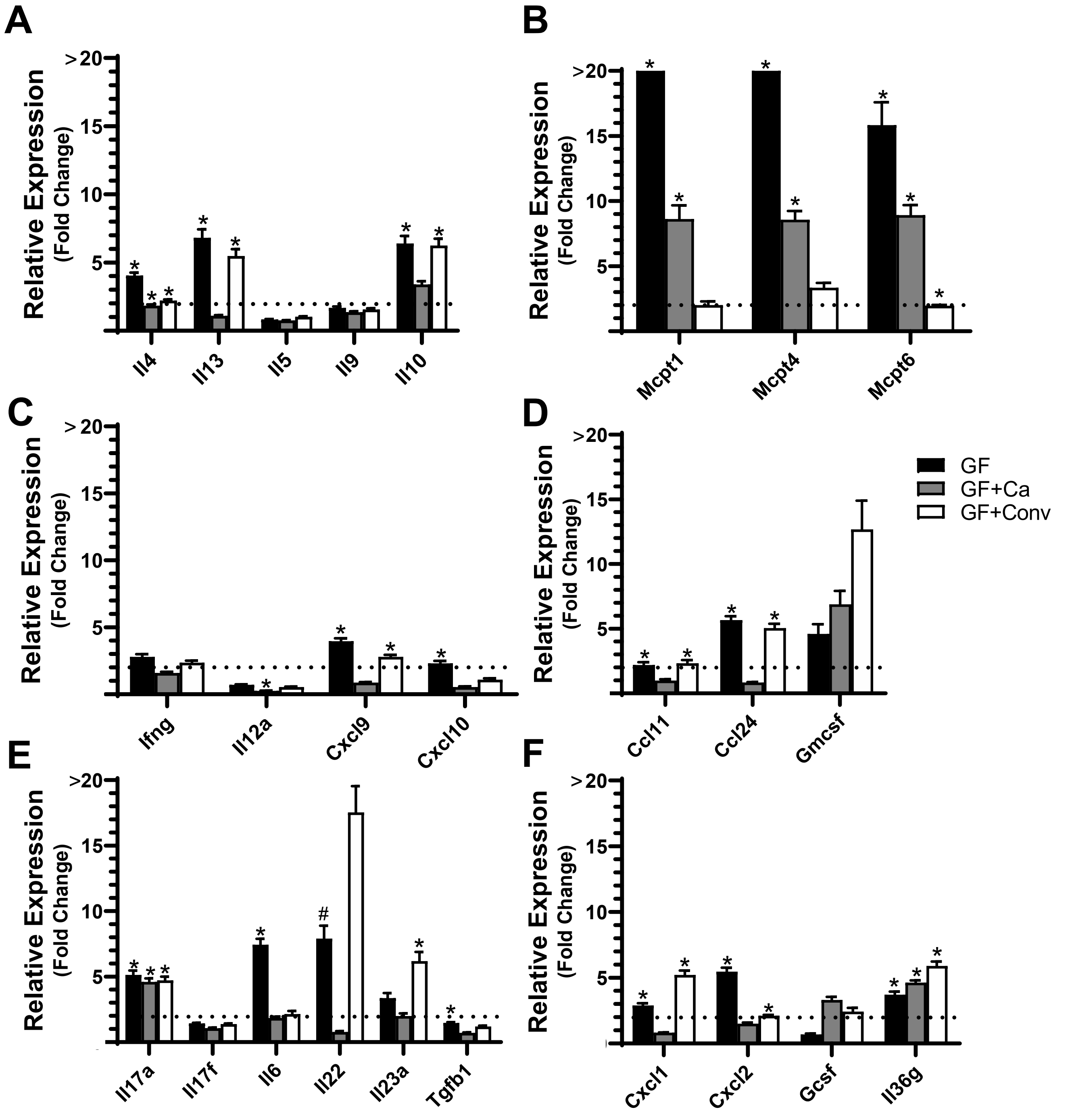

Supplemental Figure 4: Relative expression of GF, GF+Ca, and GF+Conv Balb/c mice cytokine genes in the colon after OVA sensitization and challenge (D28 or D42). Mice were treated as outlined in Figure 4A. Gene expression was measured by qPCR and is shown as relative expression compared to naïve strain-matched mice. The dotted line indicates a 2-fold upregulation from baseline expression levels. Statistical significance (*$P < 0.05$) signifies significant upregulation in the treated compared to the naïve group for each gene (treated, n = 7-10, naive, n = 5). A bar notated with a # indicates an average upregulation greater than 5 across the group, but $P > 0.05$ due to high variance in the overall dataset. Data are plotted as the mean ± SEM. Detailed description of analyses and statistical tests used can be found in the Materials and Methods. Definitions of gene abbreviations can be found in Table 1.

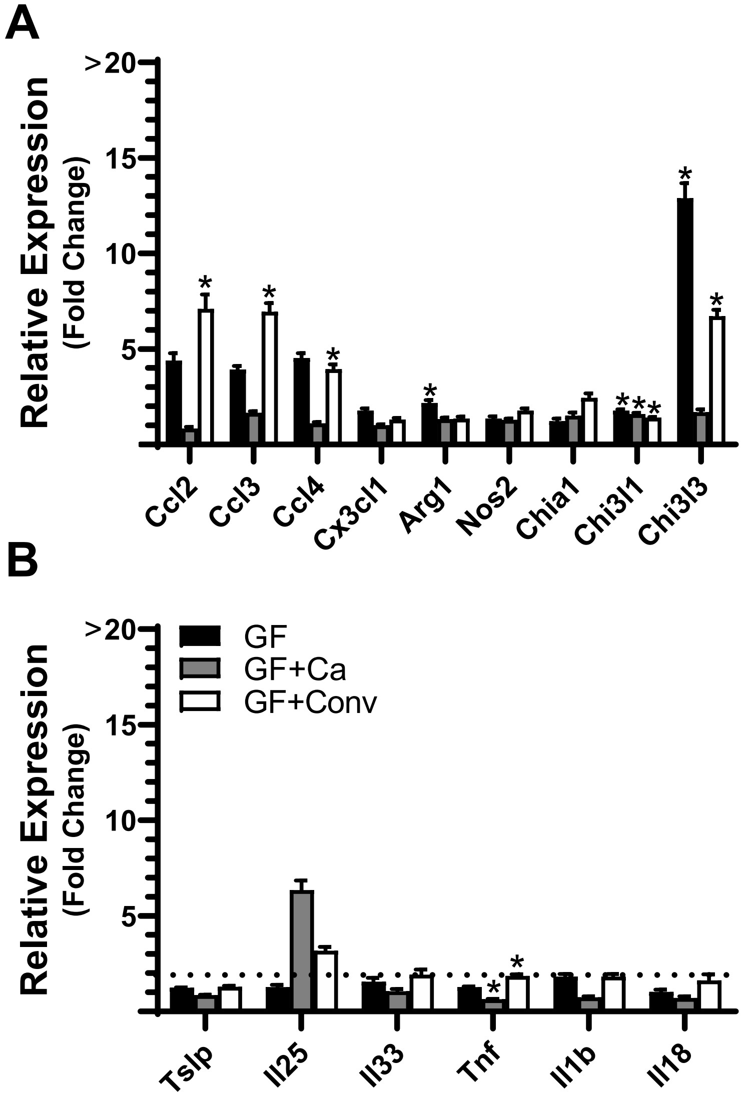

Supplemental Figure 5: Cytokine gene induction in the colon of GF, GF+Ca, and GF+Conv Balb/c mice after OVA sensitization and challenge (D28 or D42). Data shown is relative expression compared to naïve strain-matched mice. Mice were treated as outlined in Figure 4A. The dotted line indicates a 2-fold upregulation from baseline expression levels. Gene expression was measured by qPCR. Statistical significance (*$P < 0.05$) signifies significant upregulation in the treated compared to the naïve group for each gene (treated, n = 7-10, naive, n = 5). A bar notated with a # indicates an average upregulation greater than 5 across the group, but $P > 0.05$ due to high variance in the overall dataset. Data are plotted as the mean ± SEM. Detailed description of analyses and statistical tests used can be found in the Materials and Methods. Definitions of gene abbreviations can be found in Table 1.

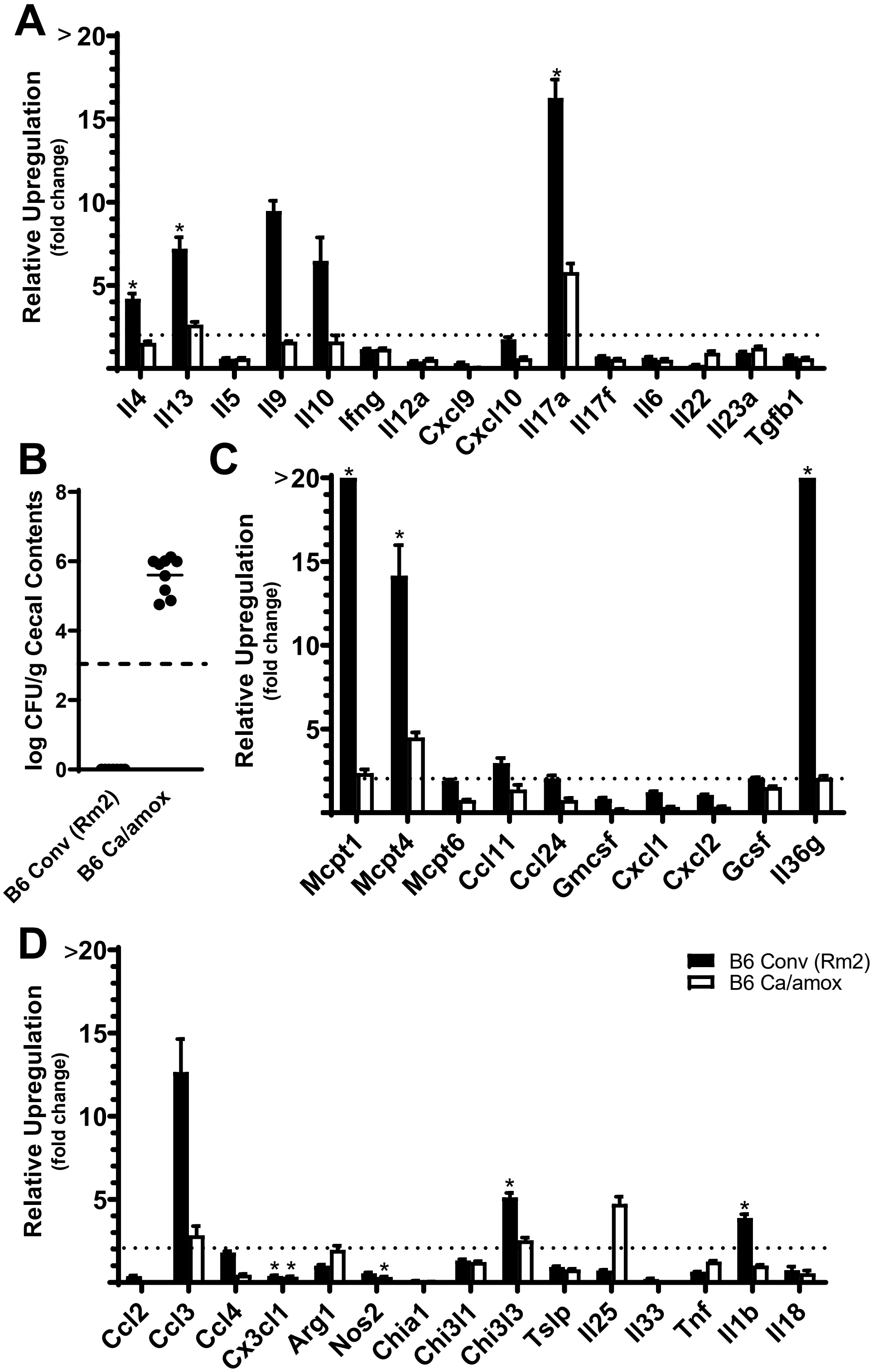

Supplemental Figure 6: Cytokine gene induction in the colon of C57BL/6J mice with a conventional microbiome (B6 Conv (Rm2)) and disrupted microbiota (B6 Ca/amox) after food allergen (OVA) sensitization and challenge (D28). **(A, C-D)** Data shown is relative expression compared to naïve strain-matched mice. Mice were treated as outlined in Figure 1A. The dotted line indicates a 2-fold upregulation from baseline expression levels. Gene expression was measured by qPCR. Statistical significance (**P* < 0.05) signifies significant upregulation in the treated compared to the naïve group for each gene (treated, *n* = 7-10, naïve, *n* = 5). A bar notated with a # indicates an average upregulation greater than 5 across the group, but *P* > 0.05 due to high variance in the overall dataset. Data are plotted as the mean ± SEM. **(B)** CFU of *C. albicans* from the cecal contents. Detailed description of analyses and statistical tests used can be found in the Materials and Methods. Definitions of gene abbreviations can be found in Table 1.

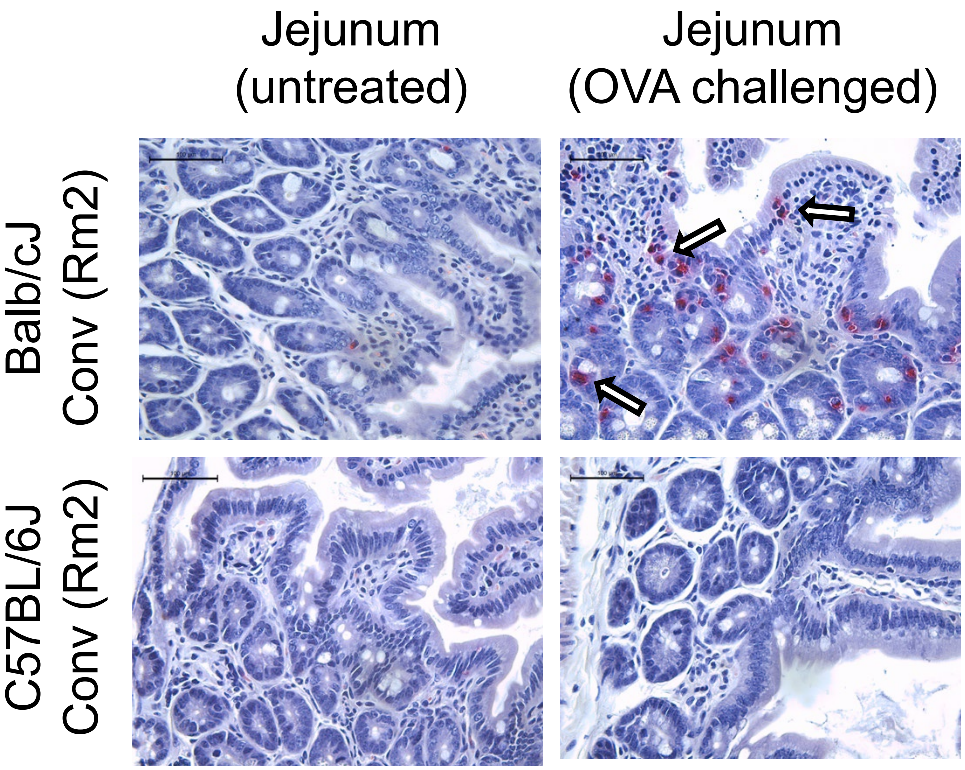

Supplemental Figure 7: Photomicrographs of representative CAE-stained sections from the small intestine of Conv (Rm2) Balb/cJ and C57BL/6J mice at Day 42. Arrows point to selected mast cells in the histological sections, as identified by chloroacetate esterase (CAE) staining (red). Note the markedly increased number of mast cells in the jejunum of Conv (Rm2) Balb/cJ mice after OVA sensitization and challenge compared to untreated Balb/cJ mice or to Conv (Rm2) C57BL/6J mice. The black ruler bar in the images is 100 mm. To prepare histological slides, tissues were fixed in 10% formalin for 24 hours and transferred to 70% ethanol prior to paraffin-embedding and sectioning. Each slide was stained with chloroacetate esterase (CAE) for visualization of intestinal MCs and counterstained with hematoxylin.
